# Human population dynamics in Upper Paleolithic Europe inferred from fossil dental phenotypes

**DOI:** 10.1126/sciadv.adn8129

**Published:** 2024-08-16

**Authors:** Hannes Rathmann, Maria T. Vizzari, Judith Beier, Shara E. Bailey, Silvia Ghirotto, Katerina Harvati

**Affiliations:** ^1^ Senckenberg Centre for Human Evolution and Palaeoenvironment, University of Tübingen, Rümelinstrasse 23, 72070 Tübingen, Germany.; ^2^ Paleoanthropology Section, Institute for Archaeological Sciences, Department of Geosciences, University of Tübingen, Rümelinstrasse 23, 72070 Tübingen, Germany.; ^3^ Department of Life Sciences and Biotechnology, University of Ferrara, Via Luigi Borsari 46, 44121 Ferrara, Italy.; ^4^ DFG Center for Advanced Studies “Words, Bones, Genes, Tools,” University of Tübingen, Rümelinstrasse 23, 72070 Tübingen, Germany.; ^5^ Department of Anthropology, New York University, 25 Waverly Place, New York, NY 10003, USA.

## Abstract

Despite extensive archaeological research, our knowledge of the human population history of Upper Paleolithic Europe remains limited, primarily due to the scarce availability and poor molecular preservation of fossil remains. As teeth dominate the fossil record and preserve genetic signatures in their morphology, we compiled a large dataset of 450 dentitions dating between ~47 and 7 thousand years ago (ka), outnumbering existing skeletal and paleogenetic datasets. We tested a range of competing demographic scenarios using a coalescent-based machine learning Approximate Bayesian Computation (ABC) framework that we modified for use with phenotypic data. Mostly in agreement with but also challenging some of the hitherto available evidence, we identified a population turnover in western Europe at ~28 ka, isolates in western and eastern refugia between ~28 and 14.7 ka, and bottlenecks during the Last Glacial Maximum. Methodologically, this study marks the pioneering application of ABC to skeletal phenotypes, paving the way for exciting future research avenues.

## Teaser

Large-scale analysis of fossil teeth unveils 40,000 years of demographic history of Ice Age hunter-gatherers in Europe.

## INTRODUCTION

Following multiple presumably short-lived dispersals of modern human hunter-gatherers out of Africa into Eurasia (*1*–*5*), the first sustained appearance of modern humans in Europe dates back to the Last Ice Age at ~45 to 50 thousand years ago (ka), marking the onset of the Upper Paleolithic (*6*–*10*). Despite extensive research from archaeological, fossil and, more recently, paleogenetic perspectives, the population history of these newcomers, who have since inhabited the European continent, remains not fully explained. The available genetic evidence from the earliest human populations, associated with the archaeologically defined Initial and Early Upper Paleolithic and Aurignacian cultural facies, suggests that they have contributed little to the gene pool of successive populations, indicating that they went largely extinct or were assimilated by subsequent dispersals (*7*, *10*–*16*). They are followed by, or merged into, a new group of people associated with the archaeologically defined Gravettian culture, a pan-European technocomplex with widespread similarities in lithic artifacts, weaponry, mortuary practices, and shared symbolic expressions (*17*, *18*). During the Gravettian, climate became increasingly cold and dry, forming open steppe environments capable of sustaining large mammal herds, which were the main subsistence resource for hunter-gatherers (*19*–*21*), and traces of complex settlements suggest a growth in population size relative to previous periods with milder climatic conditions (*6*, *19*, *22*). Despite regional variations in technology and settlement characteristics (*17*, *23*), the populations associated with the Gravettian culture have been suggested to maintain long-distance social networks across Europe (*17*, *24*, *25*) and to be biologically homogeneous, as indicated by both craniometric (*18*) and genetic evidence (*26*), although recent investigations have proposed dividing this continuum into two geographically distinct ancestry clusters (*15*).

During the second half of the Gravettian, temperatures dropped to the lowest levels of the entire Upper Paleolithic, accompanied by a rapid advance of northern hemisphere ice sheets, leading to a shift in vegetation from steppe to predominantly tundra environments, which affected the habitats of prey mammals (*20*, *27*). These changes resulted in a marked reduction of human net primary production and biomass, a decline in human population density with regional occupation hiatuses, and a breakdown of long-distance social networks (*19*, *28*–*34*). Climate deteriorations culminated in the Last Glacial Maximum (LGM) at ~26.5 to 19 ka, during which ice sheets reached their maximum extent, covering most of the northern and central European continent (*35*). Consequently, human populations in northern latitudes are hypothesized to have either gone extinct, or declined substantially in numbers, with some migrating to environmentally more favorable regions in southern glacial refugia, such as the Iberian peninsula and southern France, the Italian peninsula, the Balkans, and the southeastern European plain (*20*, *35*–*41*). Some LGM refugia might have been more favorable than others (*15*, *26*, *42*–*45*), and population extinctions, declines, and isolation events (*6*, *30*–*32*) likely led to a loss of genetic diversity (*45*), which might be reflected in distinct skeletal morphology differences between pre-and post-LGM groups (*36*, *46*–*50*).

After the LGM, temperatures steadily increased, glaciers retreated, and steppe and woodland vegetations returned, enabling the first (seasonal) reoccupations of previously abandoned areas, perhaps as early as ~19 ka (*20*, *21*, *31*, *38*, *51*). Post-LGM populations in western and central Europe, associated with the archaeologically defined Magdalenian culture, are thought to have spread either unidirectionally from the Franco-Cantabrian refugium (*6*, *15*, *52*) or bidirectionally from both western and eastern European refugia (*53*). Conversely, post-LGM populations in southern and southeastern Europe, associated with the archaeologically defined Epigravettian culture, may have expanded from the Balkans (*15*, *26*), but the origin, mode, and timing of this diffusion are not well understood. From at least ~14 ka, a lineage related to the Epigravettian culture spread across large parts of Europe and largely replaced the Magdalenian associated groups (*15*, *26*), coinciding with the first major warming period at the beginning of the Greenland Interstadial 1a-e/BøllingAllerød interstadial (*54*, *55*), although the exact dynamics of this turnover are not entirely characterized. After a short cold period, where temperatures dropped back to near-glacial conditions (*54*, *55*), the Last Ice Age ended at ~11.7 ka, and a rapid rise in temperatures marked the onset of the Early Holocene, characterized by large-scale changes in the ecosystem, including the return of forests and the replacement of cold-adapted tundra fauna by forest-dwelling animals (*56*). The genetic makeup of hunter-gatherers during the Late Glacial until Early Holocene has been proposed to be relatively homogeneous for several millennia within western and eastern Europe, with admixture between the two spheres occurring only in the late Mesolithic, perhaps partly in response to the expansion of early Neolithic farmers into Europe (*15*).

In the past two decades, paleobiological evidence derived directly from archaeological human remains has greatly advanced our understanding of European hunter-gatherer population history, both in the form of genetic (*15*, *26*, *45*) and phenotypic evidence (*18*, *36*, *47*–*50*). However, previous paleobiological investigations are generally hampered by limited datasets, with specimens sparsely and unevenly distributed across space and time, owing to the fragmentary nature of the fossil record, paired with the difficulty in extracting endogenous DNA from increasingly ancient specimens. The limitation of sample size is particularly notable for the LGM, a period of paramount importance for understanding Last Glacial population history. Moreover, although the debate revolves around complex demographic events, including bottlenecks and replacements, most previous genetic and phenotypic investigations used standard bioinformatic tools (e.g., multivariate analysis of variance, principal components analysis, clustering tools such as ADMIXTURE, and *f*-statistics) that are not well-suited to formally quantify these events (*57*, *58*). In contrast, Approximate Bayesian Computation (ABC), a powerful simulation-based statistical framework increasingly favored in population genetics, represents a robust approach to quantitatively compare and infer highly complex demographic histories through time (*59*). Conceptually, ABC involves comparing the observed genetic data to genetic variation simulated under different hypothesized evolutionary models, with the goal of identifying the best-fitting scenario among those tested. Posth *et al*. (*45*) applied ABC to mitochondrial DNA (mtDNA) data obtained from Upper Paleolithic and early Mesolithic European hunter-gatherers, which allowed them to explicitly test hypotheses regarding population bottlenecks and turnovers. However, because of the scarcity of available ancient genomes and the low resolution of single-locus genetic markers like mtDNA, their investigation was limited to a pan-European approach without considering potential geographic structuring, as suggested in later genome-wide studies (*15*).

Here, we address this research gap by compiling the largest skeletal phenotypic dataset from Ice Age Europe now available, surpassing existing genetic and phenotypic datasets. Specifically, our dataset consists of minor quasi-continuous dental variants, known as nonmetric traits (*60*), comprising information on the number and shape of cusps and roots, as well as the pattern of fissures, ridges, and grooves on tooth crowns. Dental nonmetric traits have been demonstrated to be at least moderately heritable (*61*–*63*) and, for the most part, selectively neutral (*64*–*67*), making them suitable proxies for tracking evolutionary relationships (*68*). Moreover, dental traits are ideal for illuminating ancient population history due to three practical advantages over other genetic and skeletal phenotypic markers. First, teeth are the hardest tissue in the human body, and as such, their remains are generally well preserved, even in taphonomic contexts where associated skeletal and endogenous DNA preservation is poor. Second, decades of anthropological research have generated a wealth of wellpublished dental trait data from hundreds of human fossils across the European continent, including fossils that no longer exist and for which DNA is not available (as is the case, for example, for multiple near-complete specimens from the Upper Paleolithic site at Předmostí, which were destroyed in World War II). Third, in comparison to other skeletal phenotypes, such as cranial (*18*, *36*) or limb (*47*–*50*) morphology, tooth crowns develop early during ontogeny, and their form is not altered after full formation, except by wear or pathology. As a result, data on dental traits are much more abundant than any other paleobiological data type, allowing studies to use larger samples and conduct more robust statistical analyses. We analyze our dental trait dataset with a machine learning ABC approach specifically devised for use with skeletal phenotypes, which we name Pheno-ABC. With our extensive dataset, we were able to test a range of competing demographic models of Ice Age population history with unprecedented numerical robustness, taking into account possible geographic variation by separately examining Western and Eastern Europe.

## RESULTS

### Dental dataset

We compiled a large database of geo-referenced and well-dated dental nonmetric traits from European Upper Paleolithic and early Mesolithic human skeletal remains dating to ~47 to 7 ka. All data were recorded following the reference standards of the Arizona State University Dental Anthropology System (ASUDAS). After pre-analysis treatment, which included trait dichotomization, checks for intertrait correlation, and “modified key tooth” trait counting, the dataset comprised 495 specimens, each represented by up to 39 dental traits (with an average number of observable traits per specimen of 8.52). Because our Pheno-ABC approach requires knowledge about the traits’ ancestral and derived states, we estimated the polarity for all traits included in our Ice Age database by correlating worldwide modern population trait frequencies against geographic out-of-Africa dispersal routes, and we retained only those with statistically significant estimates at α = 0.05 (table S1). This left us with a reduced dataset comprising 450 specimens, each represented by up to 20 dental traits (with an average number of observable traits per specimen of 4.61).

**Fig. 1.**
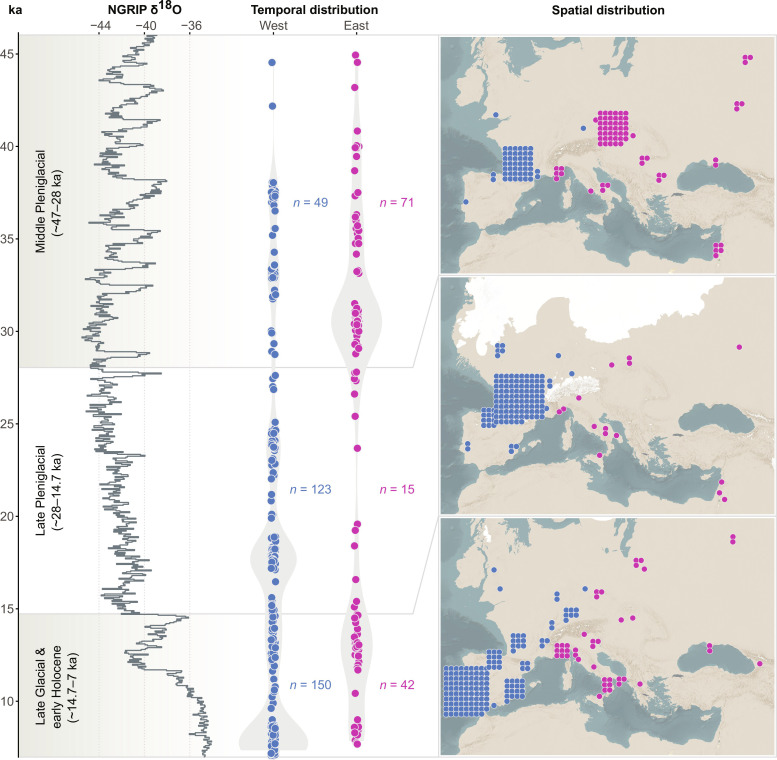
Spatiotemporal distribution of 450 European hunter-gatherer dentitions used for demographic modeling. On the left side is the North Greenland Ice Core Project (NGRIP) δ^18^O climate record (Greenland Ice Core Chronology 2005 timescale) in ‰ from 47 to 7 ka, along with three time periods whose boundaries are defined by major climatic shifts: MPG (~47 to 28 ka), LPG (~28 to 14.7 ka), and LG&EH (~14.7 to 7 ka). In the middle, the temporal distribution of specimens is presented, divided into a West group (extending from present-day Portugal to Germany) and an East group (extending from present-day Italy to Western Russia). Average calibrated radiocarbon dates are shown, with a random jitter applied to avoid overplotting. Sample sizes per spatiotemporal group are indicated. On the right, the geographical coordinates of specimens are displayed, with color-coding indicating West (blue) or East (pink) group affiliation. Geographic locations are approximated and grid-arranged to avoid overplotting. The map is temporally subdivided into MPG, LPG, and LG&EH periods, with an illustration of the advance and retreat of northern hemisphere ice sheets and associated sea level changes.

**Fig. 2.**
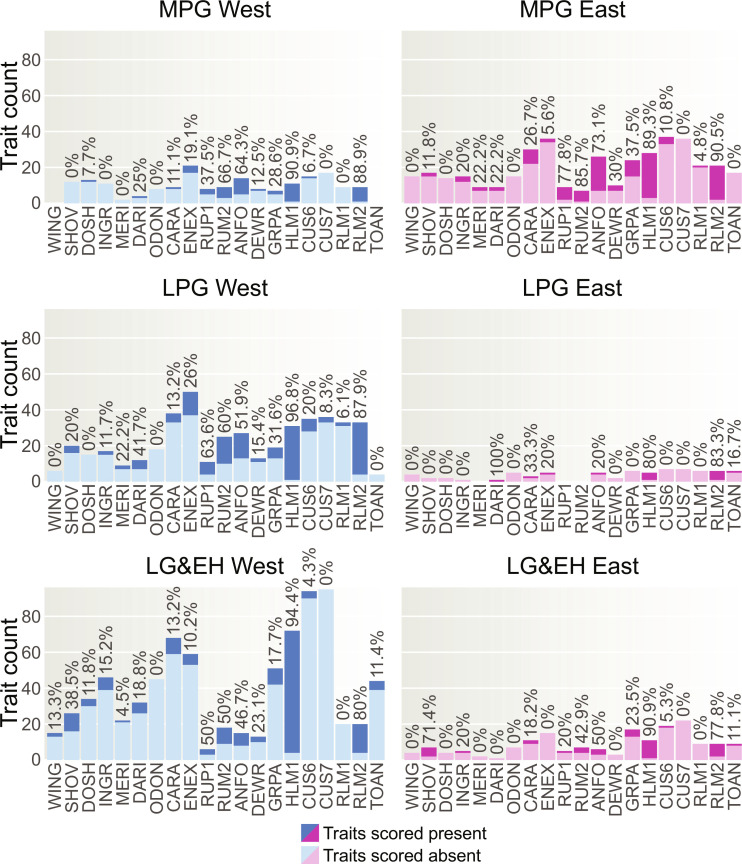
Dental trait expressions scored on 450 European hunter-gatherer dentitions divided into six spatiotemporal groups. Stacked bar plots showing absolute numbers of dichotomized absence-presence scores of 20 ASUDAS dental nonmetric traits (see table S2). In addition, the frequencies of traits scored as present is displayed. For the meaning of trait abbreviations, refer to table S1. The six groups are defined as follows: West = extending from present-day Portugal to Germany; East = extending from present-day Italy to Western Russia; MPG = Middle Pleniglacial (~47 to 28 ka); LPG = Late Pleniglacial (~28 to 14.7 ka); LG&EH = Late Glacial to Early Holocene (~14.7 to 7 ka).

Our Pheno-ABC modeling framework requires a subdivision of the data into distinct temporal sample groups. As there is growing consensus that environmental fluctuations over the course of the Last Glacial had a major impact on hunter-gatherer demography (*6*, *32*, *40*), we divided the dataset into three successive time slices whose boundaries are defined by major climatic shifts: Middle Pleniglacial (MPG; ~47 to 28 ka), Late Pleniglacial (LPG; ~28 to 14.7 ka), and Late Glacial to Early Holocene (LG&EH; ~14.7 to 7 ka). Recent research also suggests a potential population structure among huntergatherers at various periods throughout history, largely determined by their geographical distribution, with notable differences between western and eastern spheres (*15*, *30*). To consider this variation in our demographic modeling, we further divided specimens within each of the three time slices into two broad groups: West (extending from today’s Portugal to Germany) and East (extending from today’s Italy to Western Russia). Summary information about the spatiotemporal distribution of the analyzed 450 specimens is provided in Fig. 1. An overview of the dental trait expression scores recorded on these specimens within each spatiotemporal group is presented in Fig. 2 and table S2.

### Power test and model selection

**Fig. 3.**
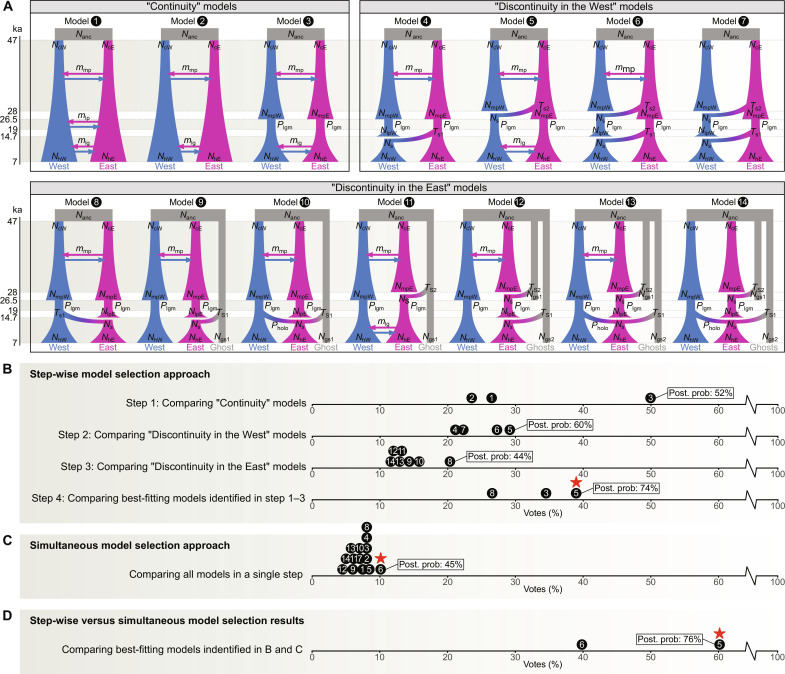
Demographic models tested and model selection results. (**A**) Schematic illustration of 14 alternative models of European hunter-gatherer population history, categorized in Continuity, Discontinuity in the West, and Discontinuity in the East. A detailed description of each model is provided in Materials and Methods. See Table 1 for demographic parameters and prior distributions. (**B**) Step-wise model selection in four steps. First, we compared the three Continuity models and identified model 3 as the best-fitting model (table S3 and fig. S1). Second, we compared the four Discontinuity in the West models and identified model 5 as the best-fitting model (table S4 and fig. S2). Third, we compared the seven Discontinuity in the East models and identified model 8 as the best-fitting model (table S5 and fig. S3). Fourth, we compared the three best-fitting models identified beforehand and identified model 5 (marked with a red star) as the best-fitting model (table S6 and fig. S4). (**C**) Simultaneous model selection considering all 14 models in a single step, identifying model 6 (marked with a red star) as the best-fitting model (table S7 and fig. S5). (**D**) Contrast between the two best-fitting models identified with the step-wise and simultaneous model selection approaches, identifying model 5 (marked with a red star) as the best-fitting model (table S8 and fig. S6).

We defined 14 alternative demographic models of European hunter-gatherer population history to be tested within our Pheno-ABC inferential framework. These models are visualized schematically in Fig. 3A. A detailed description of each model and its associated parameter prior distributions is provided in Materials and Methods. The models can be broadly subdivided into three categories.

The first category, labeled “Continuity,” includes three models (models 1 to 3). They all describe an uninterrupted population presence in both the Western and Eastern regions over time, with bidirectional migration between the two areas. The distinctions among models lie in the LPG periods, where the Western and Eastern populations are either interconnected through migration (model 1), live in isolation from each other (model 2), or go through population bottlenecks during the LGM on top of isolation (model 3).

The second category, labeled “Discontinuity in the West,” consists of four models (models 4 to 7). These models build upon model 3, yet with variations describing replacement events in the West by new incoming populations diverging from the East. The models differ in the timing of these replacement events, occurring either at the beginning of the LG&EH (model 4), at the beginning of the LPG (model 5), or at the beginnings of both the LPG and LG&EH (model 6). In addition, this category includes a variation of model 6 with no migration during the MPG (model 7).

The third category, labeled “Discontinuity in the East,” comprises seven models (models 8 to 14). They extend model 3 but with variations describing replacement events in the East by populations diverging from either the West or an unsampled external area. The timing and nature of these replacement events vary, such as occurring at the beginning of the LG&EH from either the West (model 8), from externally (model 9), or from both sources (model 10). Further variations include replacements at the beginning of the LPG from an external area (model 11), at both the beginning of the LPG and LG&EH from externally (model 12), or at the beginning of the LPG from an external source and at the beginning of the LG&EH from both the West and externally (model 13). In addition, this category includes a variation of model 13 with no migration during the MPG (model 14).

Our model comparison involves two distinct analytical procedures. At first, we conducted a power test to assess whether the models were distinguishable from each other given the dental trait data at hand, with the results expressed as classification error (CE). Then, we proceeded with the actual model selection to identify the best-fitting model, presenting the outcome as a majority vote, where the most supported model is assigned a posterior probability. In addition, we verified whether the selected model is able to generate the observed dental data by visually inspecting linear discriminant analysis (LDA) plots.

Comparing a large number of alternative models simultaneously may reduce the ABC inferential framework’s ability to accurately identify the best-fitting model, especially when the models are relatively similar to each other, as the ones used here. We therefore followed an alternative strategy, suggested to be more effective, which involves a step-wise model comparison (*69*, *70*). Under this approach, models are first compared within categories of broadly similar models. Then, the best-fitting models from these categories are compared to each other in a final contrast to find the most supported model across all models tested.

As a first step, we compared the three models within the Continuity category. When conducting the power test, we obtained a low CE for model 3 (4%), while models 1 and 2 exhibited higher errors (40 and 57%, respectively) (table S3). These high CEs stem from the similar demographic scenarios defined by models 1 and 2, with the only difference in the absence/presence of bidirectional migration between West and East during the LPG. We then performed the actual model selection, identifying model 3 as the most supported, with the highest number of votes and a posterior probability of 52% (prior expectation: 33%) (table S3 and Fig. 3B). The LDA plot confirms that model 3 is capable of generating the observed dental data (fig. S1).

Second, we compared the four models within the Discontinuity in the West category. Power test results revealed low CEs for models 4 and 5 (13 and 3%, respectively) and higher errors for models 6 and 7 (66 and 46%, respectively) (table S4). This is yet again expected given that models 6 and 7 are similar, differing only in the absence/presence of bidirectional migration between West and East during the MPG. The actual model selection, in this case, identified model 5 as the most supported, garnering the highest number of votes and being assigned a posterior probability of 60% (prior expectation: 25%) (table S4 and Fig. 3B). The LDA plot validates that model 5 is able to generate the observed variation (fig. S2).

Third, we compared the seven models within the Discontinuity in the East category. Overall, we obtained lower CEs for models 8, 9, and 11 (22, 38, and 7%, respectively) and higher errors for models 10, 12, 13, and 14 (83, 60, 77, and 70%, respectively) (table S5). The high error of model 10 stems from the fact that models 8 and 9 are nested within it, meaning that certain combinations of parameters in model 10 can produce identical demographic dynamics as those described by models 8 and 9. The high errors of models 12, 13, and 14 are expected given the similar demographic scenarios these models define, differing only in the composition of the replacing population at the onset of the LG&EH in the East on the one hand and the absence/presence of bidirectional migration between West and East during the MPG on the other. The actual model selection in this case identified model 8 as the most supported, with the highest number of votes and a posterior probability of 44% (prior expectation: 14%) (table S5 and Fig. 3B). The LDA plot confirms that model 8 can generate the observed data (fig. S3).

In the fourth and final step, we compared the three best-fitting models identified beforehand (models 3, 5, and 8) to find the most supported model across all models tested. The power test, in this case, revealed a low CE for model 8 (4%) and higher errors for models 3 and 5 (27 and 26%, respectively) (table S6). The model selection procedure in this final comparison favored model 5, assigning it the highest number of votes and a posterior probability of 74% (prior expectation: 33%) (table S6 and Fig. 3B). The LDA plot validates that model 5 is able to generate the observed dental variation (fig. S4).

Although we hold a preference for the step-wise comparison due to the reasons outlined above, we additionally performed a simultaneous comparison considering all 14 models together. Overall, the inferential power is lower compared to the results reported above, with CEs ranging from 14 to 88%, which is expected given the higher number of models compared, paired with features of some of the demographic histories that generate similar patterns of variation (table S7). In this case, the model selection procedure identified model 6 as the most supported, receiving the highest number of votes and a posterior probability of 45% (prior expectation: 7%) (table S7, Fig. 3C, and fig. S5).

To address the inconsistency between the results obtained through the step-wise and simultaneous model comparisons, we performed a direct comparison only between models 5 and 6, respectively. Power test results revealed very low CEs for both models (4 and 5%, respectively). In line with our previous step-wise comparison, the model selection again identified model 5 as the most supported, being assigned the highest number of votes and a posterior probability of 76% (prior expectation: 50%) (table S8, Fig. 3D, and fig. S6).

Last, to further assess the robustness of the available dental trait sample sizes and their impact on the obtained results, we analyzed our dataset again using the more conservative “key tooth” trait counting approach, as opposed to the previously used and more flexible modified key tooth approach. This adjustment resulted in a reduction of the dataset from 450 to 401 specimens and a decrease in the average number of observable traits per specimen from 4.61 to 3.75 (table S9). Even with this reduced dataset, our findings remain consistent, and we observe almost identical power test and model selection outcomes (tables S10 to S15), reinforcing the support for model 5.

Together, our results support model 5 as the best-fitting demographic scenario to explain our dental trait data. According to this model, populations in the West and East were connected through gene flow during the MPG. While there was population continuity in the East from the MPG to the LPG, populations in the West were replaced at the beginning of the LPG by a new incoming population diverging from the East. Subsequently, LPG populations in the West and East lived in isolation from each other without any detectable gene flow, and both underwent population bottlenecks during the LGM. Populations in both regions survived into the LG&EH, where migration resumed.

## DISCUSSION

### Modifying ABC for use with phenotypic data

When reconstructing past population structure and population history, researchers are increasingly interested in explicitly identifying the processes that generated observed patterns rather than simply presenting plausible explanations for their observations. In contemporary paleogenetic research, coalescent-based machine learning ABC has advanced to become one of the most powerful tools for comparing alternative evolutionary scenarios over time and space, taking into account natural and human-induced processes shaping demography (*71*). In this study, we adapted the ABC framework, initially developed for use with DNA markers, to dental phenotypic data, and we name this method Pheno-ABC. This opens up the exciting possibility to apply the ABC framework to archaeological time depths or regions where obtaining ancient DNA is not feasible, and where skeletal phenotypic data are the only source of information available. Furthermore, we envision that our Pheno-ABC template will stimulate a wide range of future bioanthropological applications where the explicit comparison of complex demographic scenarios based on skeletal phenotypes has previously been challenging. Dental remains appear to be ideal for this purpose, as they are not only among the best-preserved skeletal elements in the archaeological record but also conserve genetic signatures in their morphology. We reason that dental nonmetric traits, as recorded with the ASUDAS, are particularly useful for this endeavor as they fulfill many of the analytical assumptions required for ABC, namely, (i) they are at least moderately heritable (*61*–*63*); (ii) they are selectively neutral for the most part (*64*–*67*) and among the most informative morphological markers in the human skeleton (*66*); (iii) the polarity of many of the traits is known (*68*, *72*, *73*); (iv) the mutation rate can be at least approximated (see Materials and Methods); (v) they can be dichotomized into binary absence-presence scores without losing much information on trait expressivity (*74*); (vi) trait expressions are generally independent of each other (*60*, *68*); and (vii) trait expressions are not or only minimally affected by sexual dimorphism (*60*, *68*). Although already extensively researched over decades, future work should aim to further validate these fundamental assumptions, which may help attain even more robust dental trait–based Pheno-ABC model inferences in the future. Given its flexibility, we envision that the Pheno-ABC framework can, in principle, also be applied to other skeletal phenotypic nonmetric trait data commonly used in bioarchaeological research, such as cranial nonmetric traits, which may fulfill many of the required assumptions as well (*66*). An exciting area of future research should now be seeking to establish analytical ways of how to apply this framework to quantitative trait data.

### Inferring population history of Upper Paleolithic Europe from dental remains

The hunter-gatherer population history of Upper Paleolithic Europe has been widely researched using paleobiological evidence derived directly from archaeological human remains, both in the form of genetic and skeletal phenotypic data, and yet, it remains only partly understood. Questions on the mode, timing, tempo, and interplay of demographic processes persist, as only few studies have attempted to systematically coanalyze the multiple available specimens unearthed to date (*15*, *26*, *36*). The available evidence suggests several demographic transitions during the Last Glacial, which likely relate to marked climatic and environmental fluctuations and associated changes in habitat suitability and human mobility behavior (*6*, *15*, *26*, *30*, *31*, *40*), prompting sociocultural adaptations (*38*). By applying our newly developed Pheno-ABC approach to a dental dataset greatly extending now available paleobiological datasets, we were able to explicitly test a range of alternative models of European Ice Age population history with unprecedented numerical quality. Through this approach, we have generated six intriguing insights.

First, our results indicate that, during the MPG, populations in the West and East were connected through bidirectional gene flow. This finding is consistent with archaeological evidence indicating highly mobile and interconnected populations with low population sizes during the Aurignacian (*75*). Similarly, our results align with the archaeological record of the Gravettian, indicating a pan-European culture with widespread similarities in lithic technologies, weaponry, mortuary practices, shared symbolic expressions, and long-distance exchange, although being composed of a mosaic of regionally and chronologically distinct variations (*17*, *23*–*25*). Our results are also in agreement with a supposed biologically homogeneous pan-European Gravettian population, as evidenced by craniometric data (*18*). Paleogenetic investigations have suggested subdividing this Gravettian continuum into two distinct genetic ancestry clusters, the western European Fournol cluster (~32 to 21 ka, extending from Spain to France) and the eastern European Věstonice cluster (~31 to 29 ka, extending from Italy to Czechia) (*15*). Recently analyzed genomes from Buran-Kaya III in Crimea (~36 to 37 ka) assigned to the earliest appearance of the Gravettian, however, show a higher affinity with the Fournol than the Věstonice cluster (*11*). Considering that both archaeological and genetic data indicate a (partial) population turnover accompanying the transition from the Aurignacian to the Gravettian (*7*, *10*–*16*), an interesting avenue of future research could involve dividing our MPG time slice further into two respective samples when greater numbers of dental specimens from the Aurignacian are available for study.

Second, we found that populations in the West went extinct at the beginning of the LPG and were replaced by a new incoming population arriving from the East. In contrast, paleogenetic evidence suggests continuity from MPG to LPG in western Europe, attested by Solutrean-and Magdalenian-associated genomes in southern France and Spain, which share similarity with the Fournol ancestry cluster and also carry a genetic component distantly related to the Aurignacian-associated individual Goyet Q116-1 (~35 ka, Belgium) (*15*). However, a Solutrean-associated genome from Cueva del Malalmuerzo in Spain (~23 ka), not included in (*15*), shows no affinity to the preceding Věstonice cluster but instead with Goyet Q116-1; a finding that has been interpreted, among other explanations, as a possible replacement of Gravettian-associated ancestry in the West by Solutrean-associated individuals carrying Aurignacian signals (*76*). Our inferred East-to-West migration is in agreement with ~27 ka genomic evidence from Goyet in Belgium associated with the later phase of the Gravettian (and thus assigned to our LPG West group), attesting some kind of introgression of the eastern Věstonice ancestry cluster into western Europe at the beginning of the LPG (*15*). Similarly, the ~27 ka Serinyà genome from northeastern Spain shows strong affinity to the ~36 to 37 ka Buran-Kaya III genomes from Crimea (*11*). More, although not at the beginning but during the middle of the LPG, archaeological evidence suggests an east-to-west introgression of lithic industries from Epi-Aurigancian Moldova, Ukraine, and Russia (~25.5 to 23 ka) into Badegoulian France (~22.7 to 20.2 ka) (*28*, *77*), supporting the hypothesis of a possible eastern contribution to the emergence of the Badegoulian and contact between eastern and western European spheres entailing an east-to-west population expansion (*52*).

Third, we found that there was population continuity in the East from the MPG to LPG. This finding contradicts paleogenomic observations from Italy, which show a genetic turnover of the Gravettian-related Věstonice cluster by the Epigravettian-associated Villabruna cluster (~17 to 13 ka, Italy) (*15*, *26*, *42*, *45*). Our results also disagree with discontinuities observed in the archaeological record of the eastern European plains, suggesting regional fragmentation of both material culture and human occupation [including possible yet debated local settlement hiatuses (*28*, *78*, *79*)] in the Late Gravettian and during the LGM, indicating a decrease in population size and divergent cultural developments in spatiotemporally isolated regions, similar to the situation documented for the LGM in western Europe (*6*, *28*, *32*, *53*, *80*–*83*). Contrary to the early Gravettian with its large and semipermanent settlements, late Gravettian sites become smaller and more dispersed, likely representing periodic visits of highly mobile groups occasionally moving out of preferred southeastern refugia for specific resources (*81*). However, our results are in agreement with a genome from Kotias Klde Cave in Georgia (~25 ka), shown to have an ancestry predominantly shared with the MPG East genomes of Kostenki and Sunghir (~37 to 33 ka) (*84*).

Fourth, after the replacement in the West, our results suggest that over the course of the LPG, Western and Eastern populations lived in isolation from each other without any detectable gene flow, and both went through population bottlenecks during the LGM. This finding agrees with previous archaeological observations and paleodemographic modeling results, using site and radiocarbon date densities as proxies for the human presence; they suggest that LPG populations declined, probably as a result of climatic deterioration before and during the LGM, and were thus forced to migrate to environmentally more favorable but geographically distinct refugia, although the degree of genetic isolation remains debated (*20*, *30*, *32*, *34*–*41*, *85*, *86*). Our results also broadly fit with paleogenomic evidence, indicating that population contractions in glacial refugia led to reduced genetic diversity (*45*) and that Magdalenian-associated individuals of the GoyetQ2 ancestry cluster (~19 to 14 ka, extending from France to Poland) carry only a limited amount of extra affinity to the Villabruna ancestry cluster (*15*).

Fifth, we found population continuity both in the West and in the East from the LPG to the LG&EH. While this contradicts genomic evidence of a large-scale population turnover across Europe from at least ~14 ka (*15*, *26*, *42*), our inferred population continuity in the West is in partial agreement with recent genomic studies revealing that individuals belonging to the genetically defined Oberkassel ancestry cluster (~14 to 8 ka, extending from Spain to Poland) show traces (~25%) of the preceding GoyetQ2 cluster ancestry (*15*). In addition, the Solutrean-associated Cueva del Malalmuerzo lineage contributed substantially to Magdalenian-and Mesolithic-associated individuals, which attests to genetic continuity in at least southern Iberia (*76*). Our inferred population continuity in the East might be related to the fact that most of our LPG and LG&EH samples in the East group are from Italy (53 and 64%, respectively), which was dominated by the Villabruna ancestry cluster during both periods, thus drawing together the two time slices biologically. Nevertheless, and in agreement with our results, LGM genomic data from the Kotias Klde cave in the Caucasus shows that the descendants related to this ancestry contributed substantially to the formation of later West Eurasian populations (*84*). Moreover, our results agree with genomic evidence from Anatolia and the Levant, showing a high degree of continuity between ~15 ka hunter-gatherers and ~10.3 to 8.6 ka early farmers (*87*).

Sixth, we found that over the course of the LG&EH, previously isolated populations in the West and East resumed exchanging migrants. This finding broadly agrees with genomic evidence, showing that the Oberkassel ancestry cluster and the genetically defined Sidelkino ancestry cluster (~13 to 6 ka, Western Russia), although remaining relatively isolated for nearly six millennia, exchanged genes at least from ~8 ka in central Europe (*15*). Our findings are further supported by genomic data from Britain, showing that the Kendrick’s Cave individual (~13 ka) traces its ancestry to groups who expanded across Europe during the Late Glacial and are represented at sites such as Villabruna in Italy (*88*).

Together, our dentally inferred demographic scenario aligns with many aspects of available archaeological, fossil, and the most recent paleogenomic findings. Some discrepancies between our results and previous paleogenomic evidence, in particular, may stem from the different compositions of analyzed specimens regarding sample sizes as well as geographic and temporal coverage. Furthermore, our model is based on broad climatically defined periods (MPG, LPG, and LG&EH) and an artificial geographic division (West and East), which are useful for capturing the minimum complexity of past large-scale evolutionary histories but hinder direct comparability to finer-scaled archaeologically or genetically defined technocomplexes or ancestry clusters. Last, it is important to emphasize that biological variation in dental nonmetric traits should not be equated with that of genetic markers, as the nexus between genotype and phenotype remains incompletely characterized (*61*–*63*, *89*, *90*). Nevertheless, a recent study demonstrated the utility of dental nonmetric traits as an adequate proxy for SNPs in reconstructing genetic relatedness across populations, showing a strong level of correlation when assessed globally (up to *r* = 0.720, *P* < 0.001), which is even higher when assessed within a continent (up to *r* = 0.838, *P* < 0.001) (*64*), as used here.

One major limitation of this study lies in the unequal distribution of sample sizes across both space and time (Fig. 2). Although we assembled the most extensive trait dataset of Upper Paleolithic to early Mesolithic fossil dentitions to date, with relatively robust sample sizes for MPG East and LPG and LG&EH West, certain spatiotemporal groups, particularly LPG East, remained underrepresented. Furthermore, in some instances within MPG West and LPG East, dental traits were entirely unobservable. This is generally problematic as demographic inference methods, including ABC, require sufficiently large sample sizes to adequately approximate complex past demographic realities (*59*, *71*), especially when evaluating a time depth spanning several millennia. Our Pheno-ABC framework is able to explicitly account for the challenges posed by uneven sample sizes, as well as instances of entirely missing data, during the simulation stage (see Materials and Methods), although, naturally, this technical refinement cannot entirely compensate for the need for robust fossil sample sizes. Nevertheless, it is important to acknowledge that our power tests demonstrated that many of the alternative models compared, particularly those defining clearly different demographic scenarios, are highly distinguishable from each other given the available dental data at hand. In addition, the applied LDAs validated that the selected models are indeed capable of generating the observed dental data, providing further support for the robustness of our inferential framework. Last, we reanalyzed our data using a more conservative trait counting method, which reduced the number of specimens and their trait coverage, yet the results remained nearly identical, underscoring that our sample sizes are sufficient for reliably evaluating the models tested.

Future work should concentrate on including additional Upper Paleolithic and early Mesolithic dental specimens, especially from the yet underrepresented LPG time period and from Eastern Europe, particularly the Balkans and Anatolia. This may allow for testing more nuanced models, including finer temporal slices and a larger number of geographical subgroups, to better approximate complex demographic dynamics. Last, future research should aim to include not only permanent but also deciduous teeth, which may double the available dental sample size, thus further enhancing our still patchy understanding of the Last Glacial population history.

## MATERIALS AND METHODS

### Database, quality control, and pre-analysis treatment

We compiled a database of dental nonmetric traits from European Upper Paleolithic to early Mesolithic *Homo sapiens* dating between ~47 and 7 ka. Performing an extensive literature review, most data were harvested from published dental trait descriptions. To ensure comparability between published trait data collected by different observers, we restricted our compilation to trait data exclusively recorded using the ASUDAS (*60*), a widely followed reference defining a range of crown and root traits and their different rank-scale degrees of expression in the permanent dentition, ensuring a standardized scoring procedure with negligible observer error (*91*). When dental trait observations in the literature were described using a non-ASUDAS system, we instead collected the data from published images or x-ray radiographs ourselves using the ASUDAS, which has been demonstrated to be as reliable as scoring traits from original dentitions (*73*). Self-collected ASUDAS data from images were also used when no dental descriptions were reported at all or to supplement incomplete ASUDAS descriptions. When dental observations were described using a non-ASUDAS system, and there were also no images available, we transformed non-ASUDAS descriptions into ASUDAS scores, if possible. In some cases, researchers disagreed on ASUDAS scores [e.g., Brassempouy; (*92*) as opposed to (*93*)]; in these cases, we relied on the most recent published assessment. We complemented our dataset compiled from published sources with data collected from several original fossil remains. Dental specimens and references from which the data were collected are provided in data S1.

For each specimen, we recorded the geographic coordinates of the archaeological site and the chronological age, relying preferably on direct radiocarbon (14C) dates of either the skeletal specimens themselves or organic material from the immediate burial environment or find layer of the skeletal remains. 14C dates were calibrated using OxCal Online version 4.4.3 and the IntCal20 atmospheric calibration curve (*94*, *95*). If 14C dates were unavailable, then we recorded the attribution to an archaeological period as suggested by excavators and original authors, based on the stratigraphic position of the human skeletal remains and associated cultural materials. We then converted the assigned archaeological period into a calibrated calendar age using the age ranges provided in (*96*).

When recording dental traits, we followed the individual count method whereby bilaterally expressed traits were scored only once per dentition using the antimere with the highest expression level (*60*). Because of the lack of ASUDAS trait sexual dimorphism, it is standard to analyze males and females together (*60*, *68*). Rank trait expression scores were then collapsed into simplified binary dichotomies of absence (0) or presence (1) based on established breakpoints (*60*, *64*, *68*). This transformation enables the calculation of trait frequencies per spatiotemporal sample group for Pheno-ABC analysis (see below). Dichotomization removes some but not all information about variation in trait expressivity, as trait frequencies are expected to be correlated with the level of trait expressivity within a population under a threshold model of quasi-continuous variation (*74*). Moreover, dichotomized trait data hold two advantages: First, dichotomization serves as an additional quality control by further reducing any potential residual observer error (*60*, *64*, *68*). Second, heritability estimates (*h*^2^) for dichotomized traits were demonstrated to be higher than for nondichotomized traits (*61*–*63*).

Our Pheno-ABC framework is built upon the assumption of independence among dental trait expressions. To explore trait associations in our data, we estimated a tetrachoric correlation matrix for all traits, assessing the significance of pairwise trait associations with application of the Benjamini-Hochberg correction for multiple testing. In agreement with previous research (*60*, *68*), we find that traits are typically expressed independently of other traits, with only 4% of all pairwise comparisons being significant (fig. S7). Nevertheless, the same trait expressed across different members of a morphogenetic field generally shows significant correlations with one another (e.g., Cusp 6 expression on molars M1, M2, and M3 is significantly correlated). To avoid issues of trait correlation, we collapsed the dataset to traits solely recorded on one tooth per field (e.g., M1), referred to as the key tooth, considered the most stable member in terms of development and evolution (*60*, *68*). This procedure reduced the dataset to 470 specimens, each represented by up to 39 traits, with an average of 6.25 observable traits per specimen. However, given that this approach substantially reduces our dataset due to the exclusion of specimen where key teeth were not preserved, we opted for an alternative, more flexible method known as modified key tooth analysis (*97*, *98*). This method uses the key tooth in a given field (e.g., M1) unless that tooth is absent, in which case it is substituted by another tooth from the same tooth field (e.g., M2 substitutes M1) (table S1). Following this procedure, we retained a higher number of 495 specimens, once again represented by up to 39 traits, but with a higher average of 8.52 observable traits per specimen. To assess the effectiveness of both the key tooth and modified key tooth procedures in mitigating trait correlations, we recalculated tetrachoric correlations for both datasets and found, as anticipated (*60*, *68*), that the amount of significant pairwise trait associations decreased to 0.6% in the key tooth dataset (fig. S8) and 1.5% in the modified key tooth dataset (fig. S9). We deem these low amounts of intertrait correlations negligible for subsequent analysis (see below).

### Spatiotemporal subdivision of data

As our modeling framework requires distinct temporal sample groups, we divided the data into three successive time slices whose boundaries are defined by major climatic shifts: MPG (~47 to 28 ka), LPG (~28 to 14.7 ka), and LG&EH (~14.7 to 7 ka). The MPG was characterized by high-frequency and large-amplitude climate oscillations, including several cycles of cooling and warming events, resulting in a rapid advance and retreat of northern hemisphere ice sheets (*19*, *33*, *54*, *55*). Temperatures steadily declined after a last warm event at ~37 ka, with a climate similar to, or even colder than, during the subsequent LGM (*19*, *33*), favoring open steppe environments able to sustain large mammal herds (*21*). The lower bound of the MPG is broadly in compliance with the transition from the marine isotope stage (MIS) 3 to MIS 2. The LPG was characterized by drastically declining temperatures and further advancing ice sheets, resulting in a change of vegetation from steppe to predominantly tundra environments, affecting habitats of large mammal herds (*20*, *27*). In the middle of the LPG at ~26.5 to 19 ka, climate deteriorations culminated in the LGM, when ice sheets reached their maximum extent and covered most of the Northern and Central European continent (*35*). The LG&EH was characterized by the Greenland Interstadial 1a-e/Bølling-Allerød Interstadial (~14.7 to 12.9 ka), a period of rapid warming and moist conditions, followed by the Greenland Stadial 1/Younger Dryas (~12.9 to 11.7 ka), a cold period of about one millennium, where temperatures dropped back to near-glacial conditions within just decades (*54*, *55*). At ~11.7 ka, a rapid rise in temperatures marked the onset of the EH, characterized by large-scale changes in the ecosystem, including the return of forests and the replacement of cold-adapted tundra fauna by forest-dwelling animals (*56*). Although climatologically distinct, we analyzed LG and EH specimens together, as the genetic makeup of western and eastern European populations remained relatively homogeneous after ~14 ka (*15*). We assigned specimens to either of the three time slices using the mean values of age ranges.

Recent research suggests a potential population structure among Ice Age hunter-gatherers, largely determined by their geographical distribution (*15*). To account for this variation in our demographic modeling, we divided the data within each of the three time slices into two broad groups: West and East. The West group includes specimens from western Europe and regions north of the Alps, namely, today’s Portugal, Spain, France, the British Isles, the Netherlands, Belgium, Switzerland, and Germany. The East group comprises specimens from eastern Europe, the Near East, and regions south of the Alps, encompassing today’s Italy, Austria, Poland, Czechia, Slovakia, Hungary, Slovenia, Croatia, Bulgaria, Romania, Western Russia, Georgia, Lebanon, and Israel. Although simplistic, this artificial grouping broadly conforms with a suggested West-East divide of genetic ancestry during the Gravettian period, namely, the western Fournol cluster (~32 to 21 ka, from Spain to France) and the eastern Věstonice cluster (~31 to 29 ka, from Italy to Czechia) (*15*). It also aligns with a suggested environmental barrier along the 10°E longitude during the LGM, proposed to separate western and eastern populations adapting to different climatic conditions in different glacial refugia and possibly limiting interactions between them (*30*). Our grouping also generally reflects genetic evidence of the LG&EH periods, suggesting isolation between the western Oberkassel cluster (~14 to 8 ka, from Spain to Poland) and the eastern Sidelkino cluster (~13 to 6 ka, Western Russia) for nearly six millennia, with gene exchange beginning only after ~8 ka (*15*). Although further subdivision of our data into multiple spatial groups beyond West and East would be desirable for testing even more complex models of Upper Paleolithic population dynamics, the scarcity of fossil remains from certain time periods and regions and the resulting small sample sizes per subgroup ultimately limit such efforts until greater numbers of fossils become available for study. We assigned specimens to either the West or East group based on their geographic coordinates.

### Estimating ancestral and derived states for dental traits

Our Pheno-ABC approach requires knowledge about the traits’ ancestral and derived states. Assuming an origin of all modern humans in Africa, previous studies identified a dental cline in worldwide modern population trait frequencies, entailing a decrease in ancestral and an increase in derived traits from sub-Saharan Africa to North Africa, Europe, Asia, and lastly Oceania and the New World (*72*, *73*). Building upon these findings, we formally quantified the polarity of all 39 dental traits included in our Ice Age database using a large, published dataset of dental trait frequencies from worldwide modern populations and estimated the correlation between geographic distance to sub-Saharan Africa and population trait frequencies. The resulting negative/positive correlations were treated as an indicator of ancestral/derived states. Negative correlations signal a decrease in trait frequency with increasing geographic distance from sub-Saharan Africa, indicating that presence (1) is the ancestral and absence (0) is the derived state. Conversely, positive correlations signal an increase in trait frequency with increasing geographic distance from sub-Saharan Africa, indicating that absence (0) is the ancestral and presence (1) is the derived state.

Geographic distances of each population to sub-Saharan Africa were calculated as great circle distances using the Haversine and a set of most likely migration waypoints to make large-scale between-continent distances more reflective of the actual human expansion routes, avoiding large bodies of water (*99*). Our analysis comprises dental trait frequency data from 49 worldwide modern human populations, all compiled from published sources (*60*, *64*). Trait dichotomization follows the same established thresholds as used for the Ice Age trait database (*60*, *64*, *68*). In addition, to root the ancestral trait states in our modern human population samples (*100*), we integrated an outgroup of pooled Pleistocene African early *Homo* fossils, also compiled from published sources (*73*, *101*, *102*). Although ASUDAS comparisons between modern humans and archaic hominins are routinely performed and informative [e.g., (*102*)], we note that the ASUDAS was originally designed for studying modern human dental variation only, and its ability to fully capture archaic hominin variation is limited (*103*). Moreover, estimating trait states can be challenging for extinct hominins, as fossil evidence is generally scarce and, thus, ASUDAS trait frequencies are often fixed at 0 or 100%, but it is the variability in trait expression, not simply the presence or absence of such traits, that is important for determining whether a character is ancestral or derived (*72*). By pooling fossils into a larger African early *Homo* sample, we ensure more robust sample sizes. Although we note that pooling is not an ideal strategy due to spatiotemporal morphological variability among taxa, we feel this is the most viable option until greater numbers of fossil specimens are available for study.

The estimation of trait polarity was feasible for 35 of the 39 dental traits in our Ice Age dental dataset (table S1). Our estimates are in broad agreement with previous suggestions relying on worldwide trait frequencies and fossil evidence (*68*, *72*, *73*). Among these, 20 traits exhibit polarity results statistically significant at α = 0.05. We removed all traits with nonsignificant polarity estimates from our Ice Age dental dataset. This procedure reduced the key tooth dataset to 401 specimens, each represented by up to 20 traits, with an average of 3.75 observable traits per specimen. In the modified key tooth dataset, this procedure resulted in 450 specimens, again featuring up to 20 traits, albeit with a higher average of 4.61 observable traits per specimen. Details regarding dental traits per specimen in the latter dataset are provided in data S1.

### Demographic modeling with Pheno-ABC

ABC is a powerful simulation-based framework developed for comparing alternative models of evolution and inferring their parameters. Its flexibility is due to the likelihood-free inference, enabling the analysis of complex (i.e., realistic) demographic models. Under this approach, the likelihood function is approximated through simulation, incorporating prior information outlined by knowledge about the plausible values of demographic parameters that define the population dynamics under investigation. Conceptually, ABC is based on comparing the observed data, condensed to some kind of summary statistics considered to be informative, to the same set of summary statistics calculated from simulated data (*71*). The simulated data are generated through a coalescent algorithm, according to hypothesized demographic models and their parameters. The coalescent algorithm (*104*) reconstructs genealogies proceeding backward in time until reaching the most recent common ancestor of the sampled lineages through a series of coalescence events. The shape of the genealogy only depends on the demographic history tested. Mutations are then added onto the resulting tree, according to a specific rate, generating the simulated variation.

In this work, we adapted the ABC framework for use with dental traits. The rationale to do so rests on a large body of literature demonstrating that dental traits are at least moderately heritable (*61*–*63*) and evolve neutrally for the most part (*64*–*67*), thus providing adequate proxies for reconstructing evolutionary relationships (*68*). Each dental trait was treated as a genetic locus, consistent with population and quantitative genetics theory, where a heritable, additive, and neutral trait is approximately as informative about population differentiation as a single neutral genomic locus, regardless of how many loci influence the trait (*66*, *105*, *106*). ABC operates under the assumption of independence among analyzed descriptors, a premise supported by the very low amounts of intertrait correlations observed in our key tooth data (see above; figs. S8 and S9). General independence among dental trait expressions on key teeth has also been extensively demonstrated across multiple populations worldwide, at least for the most part (*60*, *68*). Nevertheless, recent studies found that some seemingly unrelated dental traits are genetically linked through pleiotropic genes, such as the ectodysplasin A receptor (*107*), although the amount of independent genetic information in dental traits appears to be high (*62*, *63*), at least in comparison to other craniodental regions (*108*–*110*). Complete independence is a common assumption made for inferences based on genomic loci, and even when not fully met, it can be considered negligible as long as the correlations are few and the magnitudes low (*111*).

Coalescent simulations require knowledge of the mutation rate, even if only in the form of a rough range. In the absence of this information for ASUDAS dental traits, we approximated this parameter by the rate of polygenic trait mutation, estimated to fall roughly between 2.6 × 10^−4^ and 5 × 10^−2^ based on experimental studies of a variety of phenotypes in different taxa (*112*). Although only a crude approximation for the mutation rate of dental traits, it aligns closely with the mutation rate of microsatellites (10^−4^ to 10^−3^) (*113*), and previous research found variation in dental nonmetric traits and microsatellites to be significantly correlated (*r* = 0.547, *P* < 0.001) (*65*). Nevertheless, future work is needed to refine the exact mutation rate for all traits listed in the ASUDAS, which may help to further reduce the variation generated by the coalescent algorithm, making power tests and model selections more robust.

In selecting an appropriate summary statistic for our observed dental data, we chose to compute trait frequencies, aligning with standard practices in dental anthropological research (*68*). We condensed the information across our entire dataset by calculating absence/presence frequencies for each trait within each of the six spatiotemporal sample groups (West and East, each further divided into MPG, LPG, and LG&EH time periods). Simulations were executed in the software ms (*114*), generating 100,000 simulations for each dental trait per demographic model. We treated each phenotypic variant as evolving independently, simulating a single polymorphic trait and calculating the frequencies of the ancestral and derived states for both West and East and in each defined time period. The simulated frequencies were computed separately for each trait and spatiotemporal group, based on the sample size available for that trait within that group. Consequently, the simulated frequencies are estimated from different sample sizes, mimicking the availability of the observed data. In instances where data for a specific trait were missing within a particular group, no simulated frequency was generated for that trait within that group. In this way, we produced simulated datasets that have the same characteristics as our observed dataset, making them directly comparable.

In its original formulation, ABC requires the simulation of millions of datasets of the same size as those observed, thus becoming computationally expensive and sometimes impractical, especially with larger datasets, a higher number of models tested, or more complex models. We, therefore, rely on a refined ABC tool based on a Random Forest (RF) machine learning algorithm (ABC-RF) (*115*). Under the RF approach, the model selection stage is rephrased as a classification problem, which reduces the number of simulations necessary to obtain reliable estimates to a few thousand. The process begins by constructing a Reference Table (RT), where each row represents a trait frequency dataset simulated from one of the alternative models alongside the index of the model that generated it. The RT then serves as the training data for the RF classifier. The RF algorithm grows an ensemble of decision trees, each of which attempts to predict the model index based on the simulated frequencies. The RF approach is well-suited for this task because it has been shown to be mostly insensitive to potentially correlated summary statistics (*115*), thereby effectively accounting for any remaining intertrait correlations in our data. Once the RF classifier is trained, it is used to predict the model index for the observed trait frequencies. This is done by passing the observed frequencies through the ensemble of trees and taking a majority classification vote among the predicted model indices. The model that received the highest number of votes corresponds to the one selected most frequently within the forest of trees, making it the best-suited choice given the observed data. There is no direct link between the number of votes across competing models and their posterior probabilities. For this reason, the support of the alternative models cannot be directly evaluated through the number of votes they received. In an ABC-RF framework, the posterior probability of the selected model identified by the highest number of votes is approximated from a secondary RF that regresses the selection error over the observed trait frequencies (*115*). All ABC-RF estimates have been obtained in R, v.3.6.3, using the package abcrf, v.1.8.1 (*115*). For the step-wise model comparisons, we used a forest consisting of 1000 trees, while for the simultaneous model comparisons, we used a forest comprising 5000 trees.

### Models

We considered a total of 14 different demographic models of European Ice Age hunter-gatherer population history, schematically illustrated in Fig. 3A. All models comprise six spatiotemporal sample groups: MPG, LPG, and LG&EH, each further divided into West and East. Although simplistic, the models are useful for capturing the minimum complexity of past large-scale evolutionary dynamics. The models can be broadly subdivided into three categories, namely, Continuity (including three models: 1 to 3), Discontinuity in the West (including four models: 4 to 7), and Discontinuity in the East (including seven models: 8 to 14). The parameter prior distributions associated with these demographic models are derived from previous studies, particularly from the work of Posth *et al*. (*45*), and are reported in Table 1. Posth *et al*. (*45*) used ABC to explore similar Upper Paleolithic population dynamics as investigated here, albeit on a pan-European scale without considering potential geographic variation. By aligning our model parameters with those used in Posth *et al*. (*45*), we ensure comparability between studies. As we are also investigating gene flow across geographical groups, a facet not examined in Posth *et al*. (*45*), we additionally relied on standard parameter prior distributions describing migration rates commonly used in genetic modeling (*116*). Following the methodology of Posth *et al*. (*45*), our models start with an ancient non-European population with a fixed effective population size, *N*_anc_, of 5000. To examine potential geographic variation within Europe, we modeled two broad populations emerging from the ancestral population and inhabiting the West and East, with effective population sizes of *N*_cW_ and *N*_cE_, respectively. We defined that these groups emerge at 47 ka, aligning with the approximate maximum age ranges of the oldest dental specimens in our dataset, on the one hand, and corresponding to the earliest assumed sustained presence of modern humans in Europe, around 45 to 50 ka, on the other (*6*–*10*). Following Posth *et al*. (*45*), we used a fixed generation time of 25 years. We drew the phenotypic mutation rate from a uniform prior distribution with a range from 2.6 × 10^−4^ to 5 × 10^−2^ (see above).

**Table 1.** Model parameter prior distributions.

Parameter	Prior	Minimum	Maximum	Reference
*N*_cW_	Loguniform	10	5000	(*45*)
*N*_cE_	Loguniform	10	5000	(*45*)
*N*_mpW_	Loguniform	500	50,000	(*45*)
*N*_mpE_	Loguniform	500	50,000	(*45*)
*N*_lpW_	Loguniform	100	50,000	(*45*)
*N*_lpE_	Loguniform	100	50,000	(*45*)
*N*_hW_	Loguniform	500	100,000	(*45*)
*N*_hE_	Loguniform	500	100,000	(*45*)
*N*_s_	Loguniform	100	50,000	(*45*)
*N*_gs1_	Loguniform	100	50,000	(*45*)
*N*_gs2_	Loguniform	100	50,000	(*45*)
*P*_lgm_	Uniform	0	1	(*45*)
*T*_s1_	Uniform	14.7 ka	19 ka	Self-defined
*T*_s2_	Uniform	28 ka	37 ka	Self-defined
*p*_holo_	Uniform	0	1	Self-defined
*m*_mp_	Loguniform	10^−6^	10^−3^	(*116*)
*m*_lp_	Loguniform	10^−6^	10^−3^	(*116*)
*m*_lg_	Loguniform	10^−6^	10^−3^	(*116*)

The first category of models, labeled Continuity, includes three scenarios (models 1 to 3), all describing an uninterrupted population presence in both Western and Eastern groups over time, with bidirectional migration between the two groups. In model 1, both groups grow exponentially up to *N*_hW_ and *N*_hE_ at 7 ka. This model includes bidirectional migration during the MPG, LPG, and LG&EH, at rates of *m*_mp_, *m*_lp_, and *m*_lg_, respectively. Model 2 mirrors model 1 but excludes bidirectional migration during the LPG, resulting in isolation between the East and West groups. Model 3 is the same as model 2, with the additional feature of a population bottleneck occurring during the LGM at 26.5 to 19 ka. In this model, the two groups grow exponentially up to *N*_mpW_ and *N*_mpE_ at 26.5 ka, followed by a reduction to constant proportions, *P*_lgm_, of their current sizes throughout the LGM. After the end of the LGM at 19 ka, both groups resume exponential growth, reaching effective sizes of *N*_hW_ and *N*_hE_ at 7 ka.

The second category of models, labeled Discontinuity in the West, consists of four scenarios (models 4 to 7), each resembling model 3 but with variations describing replacement events in the West by incoming populations diverging from the East. In model 4, the West group goes extinct at the end of the LPG, reaching a size of *N*_lpW_, and is subsequently replaced at the beginning of the LG&EH at 14.7 ka by a new incoming population diverging from the East at time *T*_s1_, with a constant size *N*_s_. Because of this influx from East LPG to West LG&EH, further modeling of bidirectional migration during the LG&EH was omitted. In model 5, the West group goes extinct at the end of the MPG, reaching a size of *N*_mpW_, and is replaced at the beginning of the LPG at 28 ka by a new incoming population diverging from the East at time *T*_s2_, with a constant size *N*_s_. Model 6 combines models 4 and 5, with the West being replaced twice, first at the beginning of the LPG and again at the beginning of the LG&EH. Model 7 is identical to model 6, with the exception that it assumes no bidirectional migration between the East and West groups during the MPG.

The third category of models, labeled Discontinuity in the East, comprises seven models (models 8 to 14), all again resembling model 3 but with variations describing replacement events in the East by populations diverging from the West or an unsampled external area. In model 8, the East group goes extinct at the end of the LPG, reaching a size of *N*_lpE_, and is subsequently replaced at the beginning of the LG&EH at 14.7 ka by a new incoming population diverging from the West at time *T*_s1_, with a constant size *N*_s_. Because of this influx from West LPG to East LG&EH, further modeling of bidirectional migration during the LG&EH was omitted. Model 9 is identical to model 8, with the difference that the new incoming population derived not from the West but from an unsampled ghost population with effective size *N*_gs1_. Model 10 combines models 8 and 9, with the new incoming population represented by an admixed group between the ghost and a proportion of the West population, *P*_holo_. In model 11, the East group goes extinct at the end of the MPG, reaching a size of *N*_mpE_, and is replaced at the beginning of the LPG at 28 ka by a new incoming population diverging from the ghost population at time *T*_s2_, with a constant size *N*_s_. Model 12 combines models 9 and 11, with the East being replaced twice, first at the beginning of the LPG and again at the beginning of the LG&EH, both by incoming populations diverging from ghost populations with effective sizes *N*_gs1_ and *N*_gs2_. Model 13 is identical to model 12, albeit with the new incoming population at the beginning of the LG&EH represented by an admixed group diverging from the ghost and West populations. Model 14 is identical to model 13, except that it assumes isolation between the East and West groups during the MPG.

### Power test and model selection procedure

When comparing models, we performed two distinct analyses: First, we evaluated the inferential power of our procedure by running a power test based on the simulated RT (*115*). For each model comparison, we treated each simulated dataset as a pseudo-observed dataset (POD), with a known demographic model of origin. We then ran the ABC-RF analysis and verified whether the POD was assigned to the model that generated it. By doing this for all the simulated datasets of the RT, we estimated the CE, representing the proportion of PODs not assigned to the true model, and the proportion of true positives (1-CE), representing the proportion of correctly assigned PODs to the true model. We also reported the confusion matrix showing how the assigned PODs are distributed among the models tested. These measures are informative about the power of the whole inferential procedure in all its various aspects, including the underlying data, model parameters used, applied data summary statistics, compared alternative models, and identification of the most supported model.

After assessing power, we performed the actual model selection applying the RF classifier trained on the simulated RT to the observed dental data. Given the large number of alternative models tested, and the fact that many of them describe evolutionary histories that are relatively similar to each other, a simultaneous comparison could potentially inflate the CE, thereby introducing bias into the RF classifier. To address this concern, we opted for an alternative strategy, which has been suggested to be more effective, namely, a step-wise model comparison (*69*, *70*). In this approach, we initially compared models within categories of broad similarity (namely, Continuity, Discontinuity in the West, and Discontinuity in the East) and identified the best-fitting model within each category. Subsequently, we conducted a final contrast by comparing the best-fitting models from these categories against each other to find the most supported model across all models tested. While we have reported the results of both the simultaneous and step-wise model comparisons, we hold a preference for the step-wise comparison due to the reasons outlined above.

To verify whether the selected models were able to generate the observed dental data, we additionally conducted an LDA, setting the flag lda = TRUE in the *R* function *abcrf* (*115*). LDA is a dimensionality reduction technique that provides a linear projection of the simulated and observed datasets on a *M-*1 dimensional space (where *M* is the number of models) that optimizes the differentiation between the models tested. We provide two-dimensional scatterplots of the first LDA axes, which offer a visual means to assess whether the simulated variation overlaps with the observed data, thereby providing information about the reliability of the models tested.
