# Supplementary material for: Human population dynamics in Upper Paleolithic Europe inferred from fossil dental phenotypes
Source: Sci Adv. 2024 Aug 16;10(33):eadn8129. doi: 10.1126/sciadv.adn8129 (PMC11328903; doi:10.1126/sciadv.adn8129)
Supplement: Supplementary file 1 — Figs S1 to S9 Tables S1 to S15 Legend for data S1 [file sciadv.adn8129_sm.pdf]

Science Advances
AAAS

Supplementary Materials for

Human population dynamics in Upper Paleolithic Europe inferred from fossil dental phenotypes

Hannes Rathmann *et al.*

Corresponding author: Hannes Rathmann, hannes.rathmann@uni-tuebingen.de; Maria T. Vizzari, vzzmtr@unife.it

Sci. Adv. **10**, eadn8129 (2024)
DOI: 10.1126/sciadv.adn8129

The PDF file includes:

Figs. S1 to S9
Tables S1 to S15
Legend for data S1

Other Supplementary Material for this manuscript includes the following:

Data S1

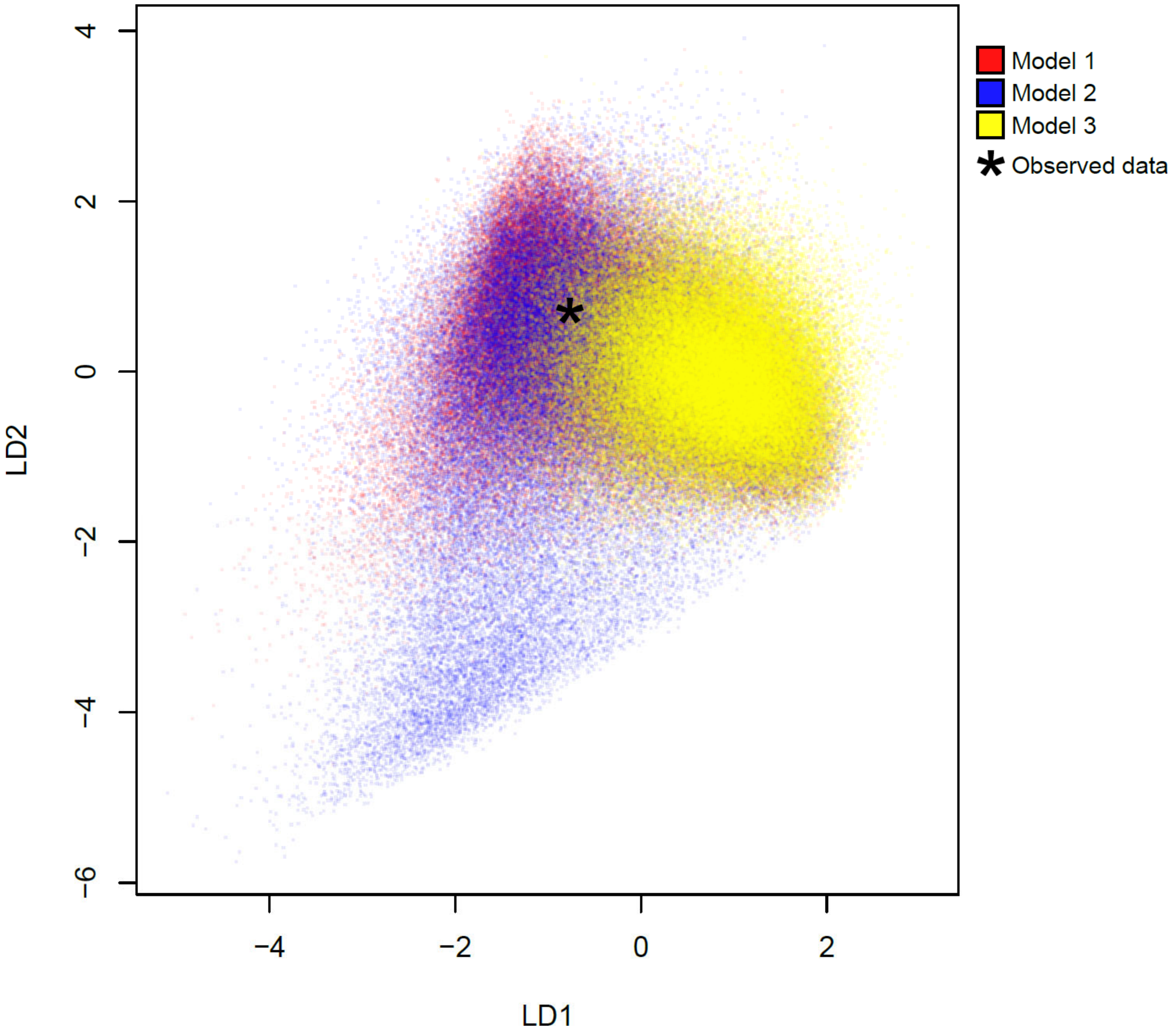

Fig. S1. LDA plot of the 'Continuity' set of models using the 'modified key tooth' dataset. Colors correspond to models. The location of the observed data is indicated by a black star.

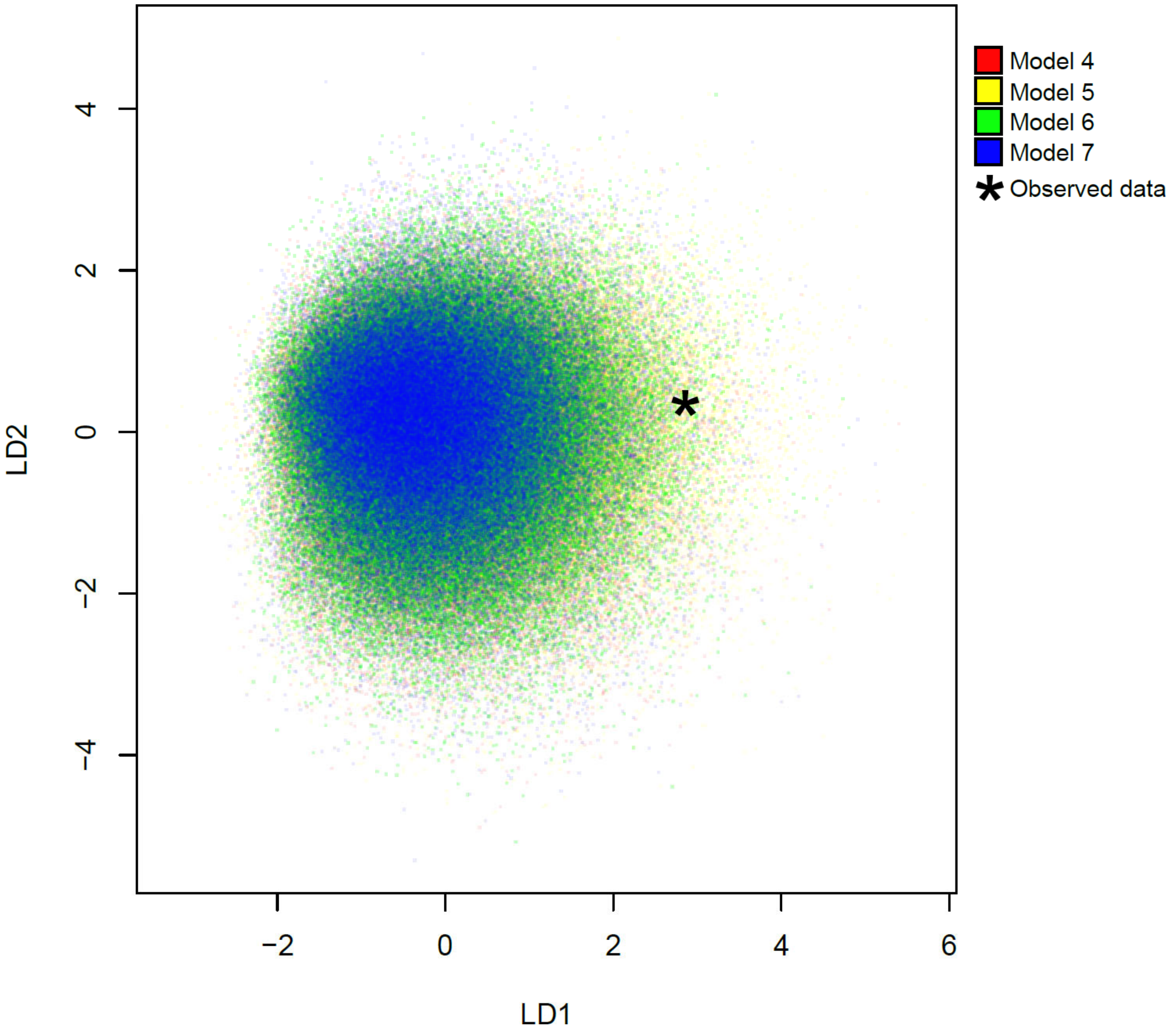

Fig. S2. LDA plot of the 'Discontinuity in the West' set of models using the 'modified key tooth' dataset. Colors correspond to models. The location of the observed data is indicated by a black star.

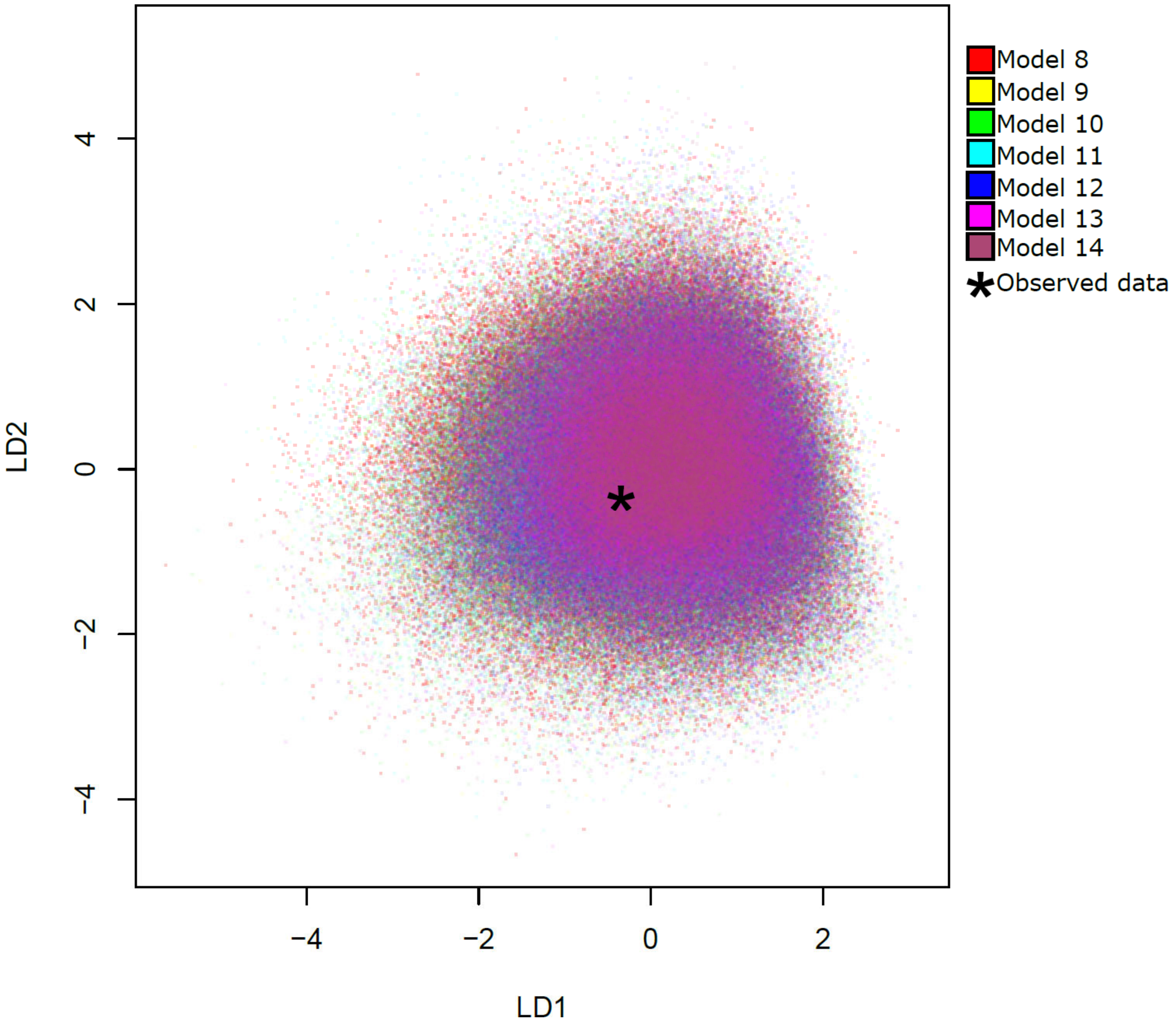

Fig. S3. LDA plot of the ‘Discontinuity in the East’ set of models using the ‘modified key tooth’ dataset. Colors correspond to models. The location of the observed data is indicated by a black star.

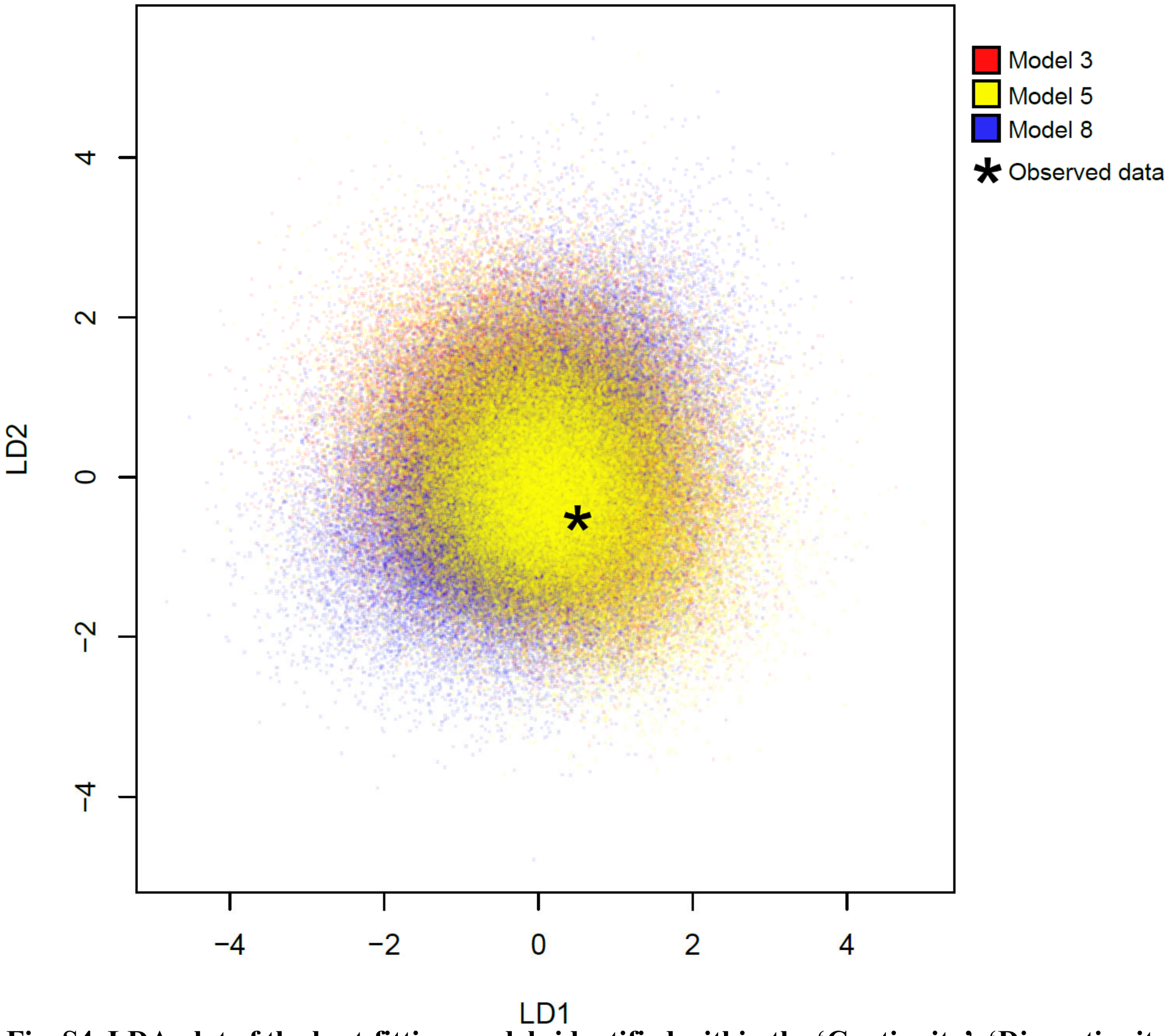

Fig. S4. LDA plot of the best-fitting models identified within the 'Continuity', 'Discontinuity in the West', and 'Discontinuity in the East' set of models using the 'modified key tooth' dataset. Colors correspond to models. The location of the observed data is indicated by a black star.

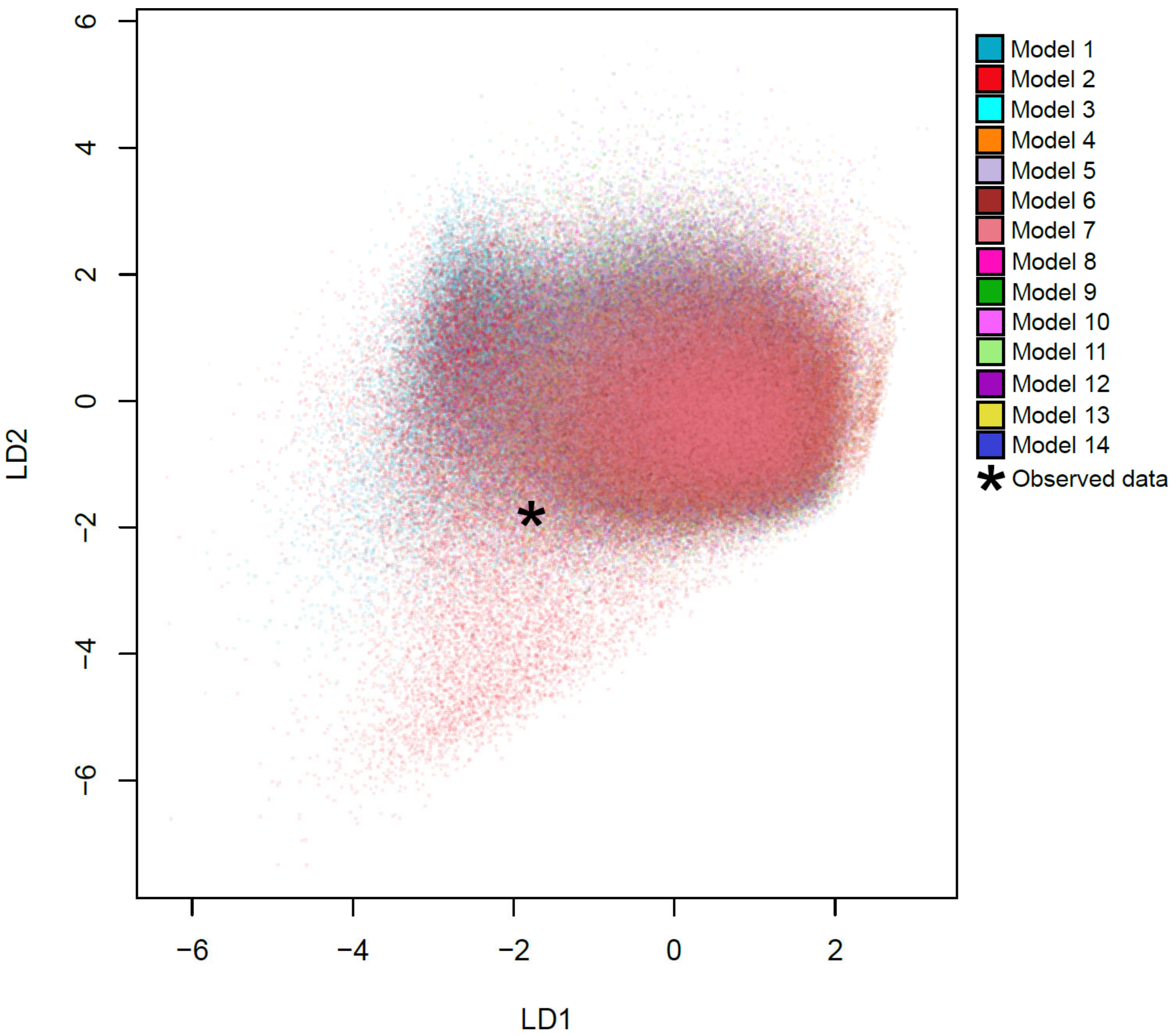

Fig. S5. LDA plot of the simultaneous model comparison using the 'modified key tooth' dataset. Colors correspond to models. The location of the observed data is indicated by a black star.

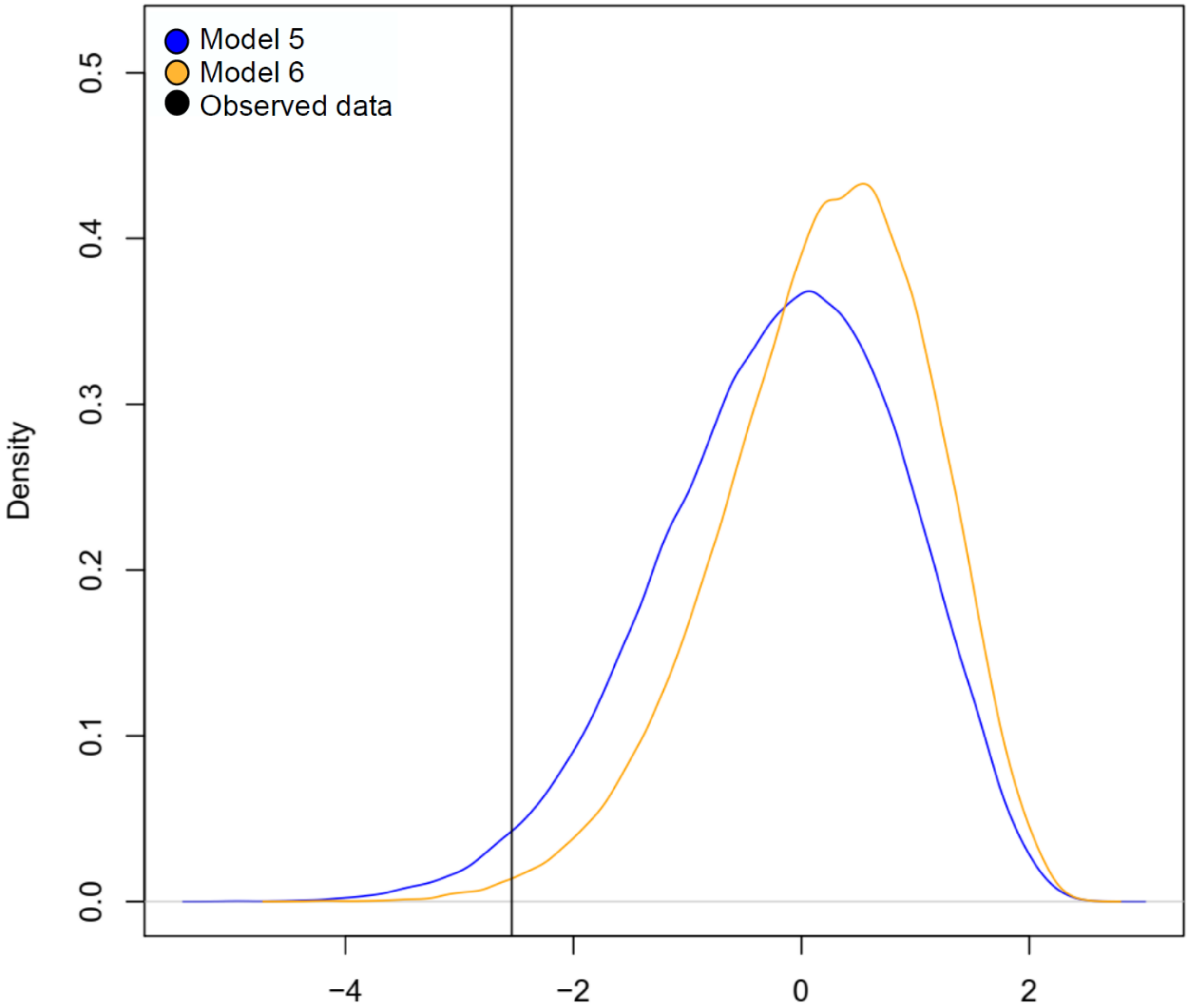

Fig. S6. LDA plot of the best-fitting models identified with the step-wise and simultaneous model selection approach using the 'modified key tooth' dataset. Colors correspond to models. The location of the observed data is indicated by a black line.

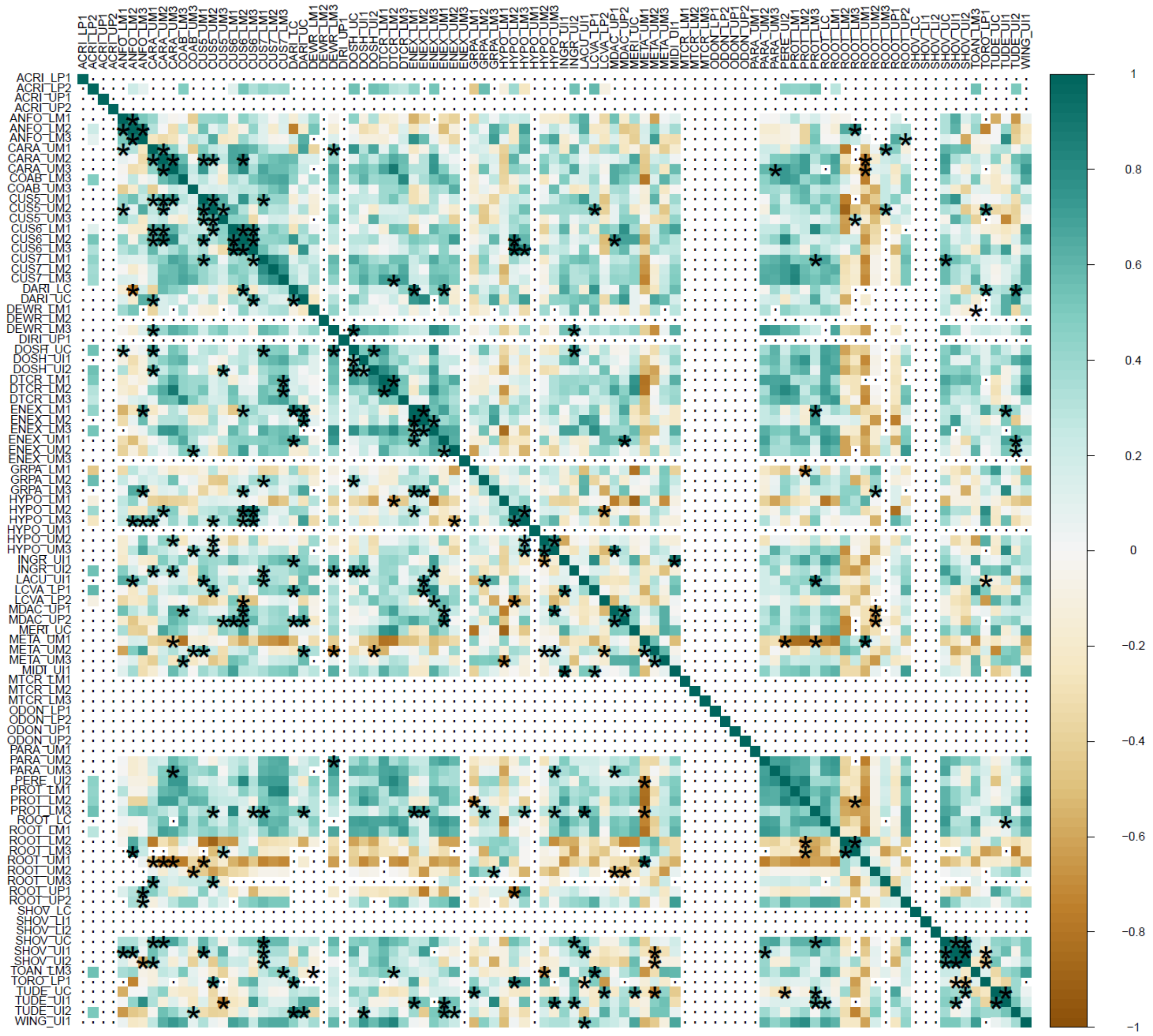

Fig. S7. Correlations among dental trait expressions in the full dataset. Presented are pairwise tetrachoric correlations, with color coding indicating the direction and strength of the correlation. Dots indicate instances where pairwise correlations could not be calculated due to insufficient data or variation. Significant associations are denoted with a black star. Significance was evaluated both without (lower triangle) and with (upper triangle) the application of the Benjamini-Hochberg correction for multiple testing. For the meaning of trait abbreviations, refer to Table S1. Out of 1,781 estimated pairwise correlations, 72 remain significant after the Benjamini-Hochberg correction, resulting in an overall ~4% significant trait associations in the dataset.

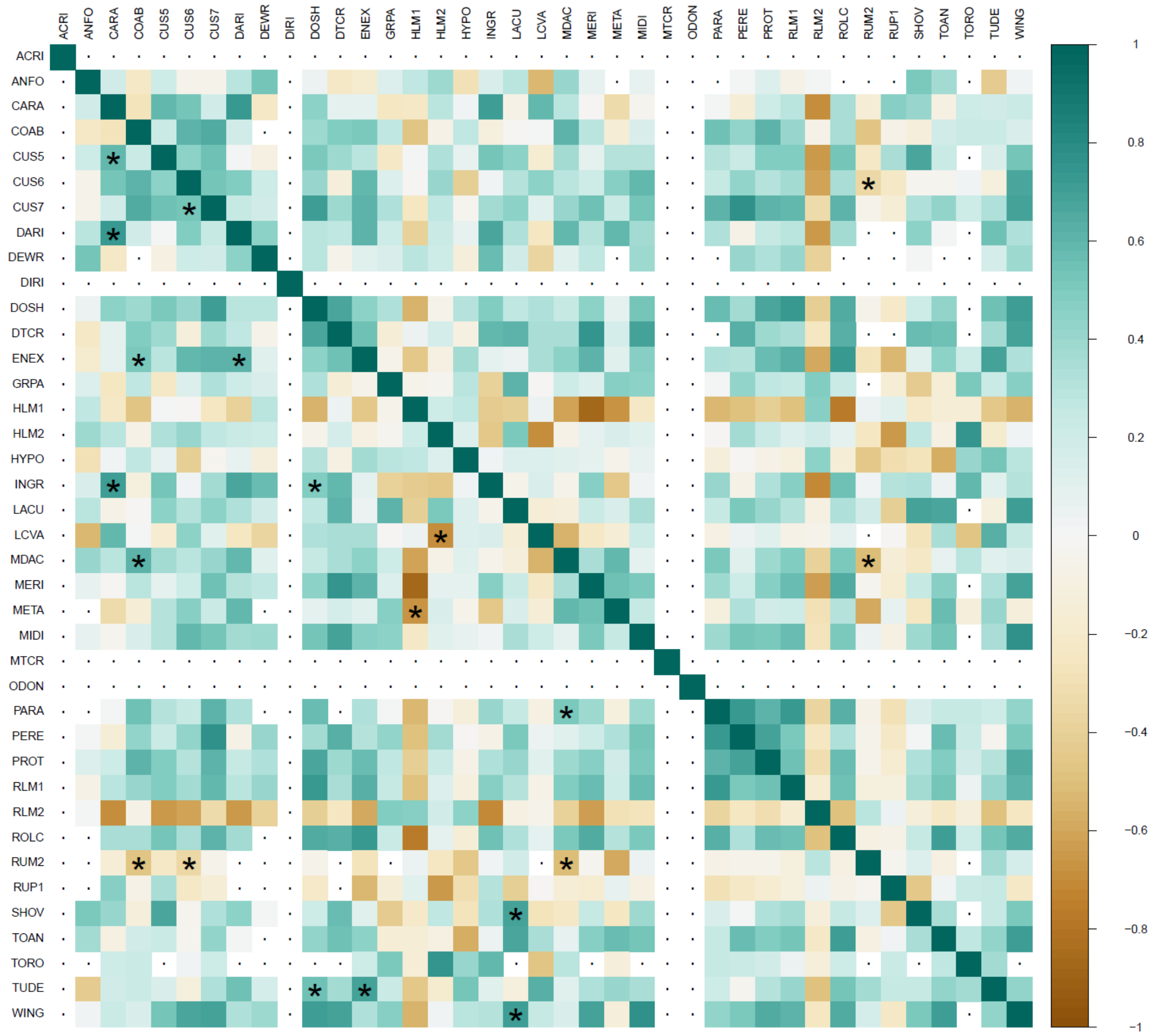

Fig. S8. Correlations among dental trait expressions in the 'key tooth' dataset. Presented are pairwise tetrachoric correlations, with color coding indicating the direction and strength of the correlation. Dots indicate instances where pairwise correlations could not be calculated due to insufficient data or variation. Significant associations are denoted with a black star. Significance was evaluated both without (lower triangle) and with (upper triangle) the application of the Benjamini-Hochberg correction for multiple testing. For the meaning of trait abbreviations, refer to Table S1. Out of 336 estimated pairwise correlations, two remain significant after the Benjamini-Hochberg correction, resulting in an overall ~0.6% significant trait associations in the dataset.

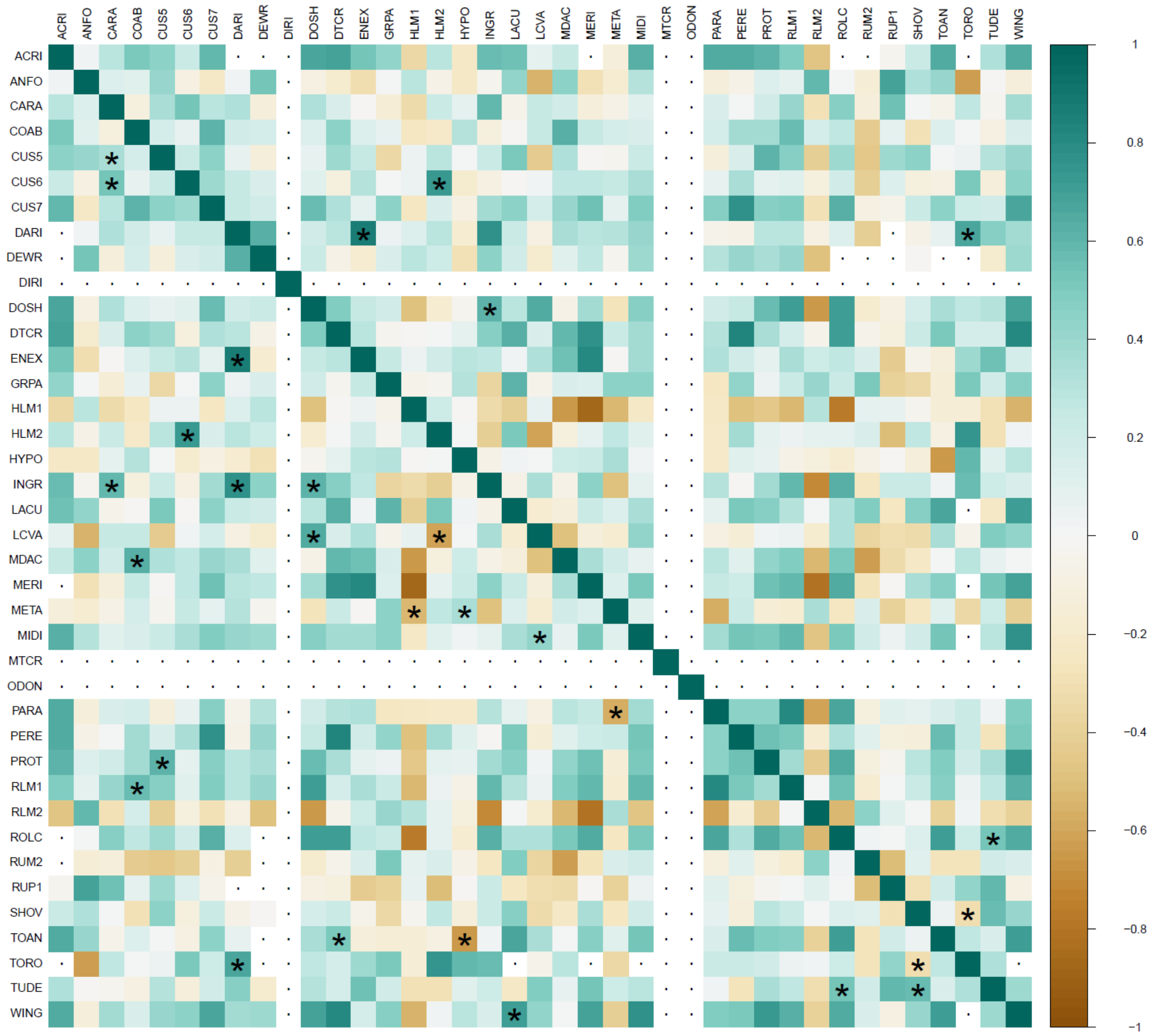

Fig. S9. Correlations among dental trait expressions in the 'modified key tooth' dataset. Presented are pairwise tetrachoric correlations, with color coding indicating the direction and strength of the correlation. Dots indicate instances where pairwise correlations could not be calculated due to insufficient data or variation. Significant associations are denoted with a black star. Significance was evaluated both without (lower triangle) and with (upper triangle) the application of the Benjamini-Hochberg correction for multiple testing. For the meaning of trait abbreviations, refer to Table S1. Out of 406 estimated pairwise correlations, six remain significant after the Benjamini-Hochberg correction, resulting in an overall ~1.5% significant trait associations in the dataset.

Table S1. Description of dental traits used in this study. Dental non-metric traits follow the ASUDAS nomenclature, with anatomical descriptions provided in ref. (60). Key tooth abbreviations denote the type of tooth scored for a particular trait, following ref. (60): C=canine; I=incisor; L=lower mandibular dentition; M=molar; P=premolar; U=upper maxillary dentition; Numbers refer to tooth positioning. The modified key tooth scoring strategy follows ref. (97, 98), utilizing the key tooth in a given field (e.g., M1) unless that tooth is absent, in which case it is substituted by another tooth from the same tooth field (e.g., M2 substitutes M1). Dichotomization breakpoints follow ref. (60, 64, 68), indicating thresholds for collapsing ordinal-ranked trait scales into binary categories of absent vs. present. Ancestral vs. derived trait states are estimated through Pearson correlation, comparing published modern population trait frequencies worldwide with geographic distance to Sub-Saharan Africa. Negative correlations indicate decreasing trait frequency with increasing distance, suggesting presence is ancestral and absence is derived. Conversely, positive correlations indicate increasing trait frequency with increasing distance, suggesting absence is ancestral and presence is derived. 'No data' indicates the inability to calculate Pearson correlation due to the absence of published worldwide modern population trait frequencies.

Dental non-metric trait (abbreviation)	Key tooth	Modified key tooth	Dichotomization breakpoints		Ancestral vs. derived				
			Absent	Present	r	n	t	df	p
Winging (WING)	UI1	UI1	2,3,4	1	0.6059	50	5.2773	48	<0.0001
Midline diastema (MIDI)	UI1	UI1	0	1	-0.0550	12	0.1743	10	0.8650
Labial curvature (LACU)	UI1	UI1	0-1	2-5	-0.4674	13	1.7539	11	0.1072
Shoveling (SHOV)	UI1	UI1 < UI2	0–2	3–7	0.6138	50	5.3874	48	<0.0001
Double shoveling (DOSH)	UI1	UI1 < UI2	0–1	2–6	0.4968	50	3.9662	48	0.0002
Interruption groove (INGR)	UI2	UI2 < UI1	0	m,d,md,med	0.4625	50	3.6143	48	0.0007
Tuberculum dentale (TUDE)	UI2	UI2 < UI1 < UC	0-1	2-6	-0.0164	49	0.1129	47	0.9105
Mesial ridge (MERI)	UC	UC	0	1-3	-0.4940	50	3.9367	48	0.0002
Distal accessory ridge (DARI)	UC	UC < LC	0-1	2-5	0.7493	26	5.5434	24	<0.0001
Mesial and Distal Accessory Cusps (MDAC)	UP1	UP1 < UP2	0	1-3	No data				
Accessory Ridges (ACRI)	UP2	UP2 < UP1 < LP1 < LP2	0-1	2-4	No data				
Distosagittal Ridge (DIRI)	UP1	UP1	0	1	0.1483	11	0.4500	9	0.6633
Odontome (ODON)	P1-P2	P1-P2			0.3641	50	2.7087	48	0.0093
Metacone (META)	UM3	UM3 < UM2 < UM1	0-3	3.5-5	No data				
Hypocone (HYPO)	UM2	UM2 < UM3 < UM1	0-2	3-6	0.1309	50	0.9149	48	0.3648
Cusp 5 (CUS5)	UM1	UM1 < UM2 < UM3	0-1	2-5	0.2719	49	1.9372	47	0.0587
Carabelli's trait (CARA)	UM1	UM1 < UM2 < UM3	0-2	3-7	-0.4576	50	3.5663	48	0.0008
Parastyle (PARA)	UM3	UM3 < UM2 < UM1	0-2	3-6	-0.0333	48	0.2265	46	0.8217
Enamel extension (ENEX)	UM1	UM1 < UM2 < UM3 < LM1 < LM2 < LM3	0	1-3	0.7162	50	7.1101	48	<0.0001
Root number (RUP1)	UP1	UP1 < UP2	1	2-3	-0.6714	50	6.2777	48	<0.0001
Root number (RUM2)	UM2	UM2 < UM3 < UM1	1-2	3	-0.4777	50	3.7677	48	0.0004
Peg-reduced (PERE)	UI2	UI2	0	P,R	-0.1784	12	0.5734	10	0.5789
Congenital absence (COAB)	UM3	UM3 < LM3	0,P,R	M	0.2241	50	1.5938	48	0.1175
Lingual cusp variation (LCVA)	LP2	LP2 < LP1	0-1	2-9	-0.2018	50	1.4276	48	0.1598
Anterior fovea (ANFO)	LM1	LM1 < LM2 < LM3	0-1	2-4	-0.8159	13	4.6804	11	0.0006

Deflecting wrinkle (DEWR)	LM1	LM1	0-1	2-3	0.5700	25	3.3275	23	0.0029
Groove pattern (GRPA)	LM2	LM2	x,+	y	-0.5123	50	4.1339	48	0.0001
Distal trigonid crest (DTCR)	LM1	LM1 < LM2 < LM3	0	1	0.2352	24	1.1352	22	0.2685
Mid trigonid crest (MTCR)	LM1	LM1 < LM2 < LM3	0	1	No data				
Protostylid (PROT)	LM1	LM1 < LM2 < LM3	0-2	3-7	0.1132	48	0.7727	46	0.4436
Hypoconulid (HLM1)	LM1	LM1	0	1-5	0.4900	17	2.1772	15	0.0458
Hypoconulid (HLM2)	LM2	LM2	0	1-5	0.1779	50	1.2530	48	0.2162
Cusp 6 (CUS6)	LM1	LM1 < LM2 < LM3	0	1-5	0.7964	50	9.1258	48	<0.0001
Cusp 7 (CUS7)	LM1	LM1 < LM2 < LM3	0-1	2-4	-0.4762	50	3.7519	48	0.0004
Root number (ROLC)	LC	LC	1	2	-0.1915	50	1.3521	48	0.1826
Root number (RLM1)	LM1	LM1 < LM2 < LM3	1-2	3	0.3170	50	2.3162	48	0.0248
Root number (RLM2)	LM2	LM2 < LM3 < LM1	1	2	-0.3822	50	2.8662	48	0.0061
Tome's root (TORO)	LP1	LP1	0-2	3-5	0.1148	49	0.7925	47	0.4320
Torsomolar angle (TOAN)	LM3	LM3	0	1	0.7419	13	3.6705	11	0.0036

Table S2. Dental trait sample sizes for the 'modified key tooth' dataset. Shown are 20 ASUDAS dental non-metric traits, the observable trait sample size (n), the number of traits scored as absent (–), the number of traits scored as present (+), and the frequencies of traits scored as present (%) for six spatiotemporal groups defined for demographic modeling. For the meaning of trait abbreviations, refer to Table S1. 'NA' indicates the inability to calculate trait frequencies because the trait could not be observed in the spatiotemporal group. The six groups are defined as follows: West=extending from present-day Portugal to Germany; East=extending from present-day Italy to Western Russia; MPG=Middle Pleniglacial (~47–28 kya); LPG=Late Pleniglacial (~28–14.7 kya); LG&EH=Late Glacial to Early Holocene (~14.7-7 kya).

Trait	West MPG				West LPG				West LG&EH				East MPG				East LPG				East LG&EH			
	n	-	+	%	n	-	+	%	n	-	+	%	n	-	+	%	n	-	+	%	n	-	+	%
WING	0	0	0	NA	6	6	0	0.00	15	13	2	13.33	15	15	0	0.00	4	4	0	0.00	4	4	0	0.00
SHOV	12	12	0	0.00	20	16	4	20.00	26	16	10	38.46	17	15	2	11.76	2	2	0	0.00	7	2	5	71.43
DOSH	13	12	1	7.69	15	15	0	0.00	34	30	4	11.76	14	14	0	0.00	2	2	0	0.00	4	4	0	0.00
INGR	11	11	0	0.00	17	15	2	11.76	46	39	7	15.22	15	12	3	20.00	1	1	0	0.00	5	4	1	20.00
MERI	2	2	0	0.00	9	7	2	22.22	22	21	1	4.55	9	7	2	22.22	0	0	0	NA	2	2	0	0.00
DARI	4	3	1	25.00	12	7	5	41.67	32	26	6	18.75	9	7	2	22.22	1	0	1	100.00	1	1	0	0.00
ODON	8	8	0	0.00	18	18	0	0.00	45	45	0	0.00	15	15	0	0.00	5	5	0	0.00	7	7	0	0.00
CARA	9	8	1	11.11	38	33	5	13.16	68	59	9	13.24	30	22	8	26.67	3	2	1	33.33	11	9	2	18.18
ENEX	21	17	4	19.05	50	37	13	26.00	59	53	6	10.17	36	34	2	5.56	5	4	1	20.00	15	15	0	0.00
RUP1	8	5	3	37.50	11	4	7	63.64	6	3	3	50.00	9	2	7	77.78	0	0	0	NA	5	4	1	20.00
RUM2	9	3	6	66.67	25	10	15	60.00	18	9	9	50.00	7	1	6	85.71	0	0	0	NA	7	4	3	42.86
ANFO	14	5	9	64.29	27	13	14	51.85	15	8	7	46.67	26	7	19	73.08	5	4	1	20.00	6	3	3	50.00
DEWR	8	7	1	12.50	13	11	2	15.38	13	10	3	23.08	10	7	3	30.00	2	2	0	0.00	3	3	0	0.00
GRPA	7	5	2	28.57	19	13	6	31.58	51	42	9	17.65	24	15	9	37.50	6	6	0	0.00	17	13	4	23.53
HLM1	11	1	10	90.91	31	1	30	96.77	72	4	68	94.44	28	3	25	89.29	5	1	4	80.00	11	1	10	90.91
CUS6	15	14	1	6.67	35	28	7	20.00	94	90	4	4.26	37	33	4	10.81	7	7	0	0.00	19	18	1	5.26
CUS7	17	17	0	0.00	36	33	3	8.33	95	95	0	0.00	36	36	0	0.00	7	7	0	0.00	22	22	0	0.00
RLM1	9	9	0	0.00	33	31	2	6.06	20	20	0	0.00	21	20	1	4.76	6	6	0	0.00	9	9	0	0.00
RLM2	9	1	8	88.89	33	4	29	87.88	20	4	16	80.00	21	2	19	90.48	6	1	5	83.33	9	2	7	77.78
TOAN	0	0	0	NA	4	4	0	0.00	44	39	5	11.36	17	17	0	0.00	6	5	1	16.67	9	8	1	11.11

Table S3. Confusion matrix and model selection results for the 'Continuity' set of models using the 'modified key tooth' dataset. The most supported model is highlighted in bold.

Confusion Matrix						Model Selection	
	Model 1	Model 2	Model 3	Classification Error (%)	True Positives (%)	Votes	Posterior Probability
Model 1	59623	29904	10473	40.38	59.62	265	-
Model 2	46613	43323	10064	56.68	43.32	236	-
**Model 3**	**2054**	**2184**	**95762**	**4.24**	**95.76**	**499**	**52%**

Table S4. Confusion matrix and model selection results for the ‘Discontinuity in the West’ set of models using the ‘modified key tooth’ dataset. The most supported model is highlighted in bold.

Confusion Matrix							Model Selection	
	Model 4	Model 5	Model 6	Model 7	Classification Error (%)	True Positives (%)	Votes	Posterior Probability
Model 4	86762	2521	7752	2965	13.24	86.76	212	-
**Model 5**	**326**	**96641**	**1382**	**1651**	**3.34**	**96.66**	**292**	**60%**
Model 6	17970	8023	33981	40026	66.01	33.99	273	-
Model 7	10458	8122	27352	54068	45.92	54.08	223	-

Table S5. Confusion matrix and model selection results for the 'Discontinuity in the East' set of models using the 'modified key tooth' dataset. The most supported model is highlighted in bold.

Confusion Matrix										Model Selection	
	Model 8	Model 9	Model 10	Model 11	Model 12	Model 13	Model 14	Classification Error (%)	True Positives (%)	Votes	Posterior Probability
**Model 8**	**77948**	**2532**	**7543**	**2605**	**610**	**3923**	**4839**	**22.35**	**77.65**	**203**	**44%**
Model 9	8358	61455	10751	5244	9212	2119	2861	38.19	61.81	143	-
Model 10	38551	27095	17069	3932	4136	4144	5073	82.93	17.07	158	-
Model 11	454	1408	1144	92530	1913	971	1580	7.25	92.75	132	-
Model 12	2878	17864	3229	15026	39019	10621	11363	60.30	39.70	120	-
Model 13	15789	7643	4821	10813	16604	23604	20726	76.89	23.11	128	-
Model 14	15732	8065	4750	10453	14061	17259	29680	70.37	29.63	116	-

Table S6. Confusion matrix and model selection results for the final comparison among the three best-fitting models using the ‘modified key tooth’ dataset. The most supported model is highlighted in bold.

Confusion Matrix						Model Selection	
	Model 3	Model 5	Model 8	Classification Error (%)	True Positives (%)	Votes	Posterior Probability
Model 3	73373	22459	4168	26.63	73.37	345	-
**Model 5**	**21882**	**74048**	**4070**	**25.95**	**74.05**	**389**	**74%**
Model 8	1834	1813	96353	3.65	96.35	266	-

Table S7. Confusion matrix and model selection results for the simultaneous model comparison using the 'modified key tooth' dataset. The most supported model is highlighted in bold.

	**Confusion Matrix**																**Model Selection**	
	Model 1	Model 2	Model 3	Model 4	Model 5	Model 6	Model 7	Model 8	Model 9	Model 10	Model 11	Model 12	Model 13	Model 14	Classification Error (%)	True Positives (%)	Votes	Posterior Probability
Model 1	60642	30855	3003	573	552	497	566	295	736	489	590	487	208	507	39.36	60.64	373	-
Model 2	48373	43005	3036	504	596	455	536	210	748	396	777	694	190	480	57.00	43.01	402	-
Model 3	3340	4023	40879	2880	18021	1832	1285	1010	2992	1547	18826	1718	688	959	59.12	40.88	402	-
Model 4	23	30	240	86303	540	8350	3569	134	135	133	219	91	96	137	13.70	86.30	401	-
Model 5	228	403	9238	491	65005	3423	5030	705	1536	990	10628	1097	632	594	35.00	65.01	417	-
**Model 6**	**14**	**48**	**162**	**22131**	**3850**	**33086**	**39707**	**168**	**120**	**161**	**169**	**104**	**134**	**146**	**66.91**	**33.09**	**505**	**45%**
Model 7	30	49	120	14217	4207	27781	52662	148	142	131	142	100	103	168	47.34	52.66	358	-
Model 8	815	1326	620	1489	1398	1282	939	79152	1561	4066	332	573	2678	3769	20.85	79.15	399	-
Model 9	1315	2192	3530	1579	2379	1163	848	7872	55308	8004	1084	9976	1880	2870	44.69	55.31	304	-
Model 10	1089	1823	2065	1518	2337	1298	914	40546	22980	11957	689	4384	3504	4896	88.04	11.96	345	-
Model 11	1117	2236	14627	2166	11279	1627	1006	331	1102	466	57443	3184	1201	2215	42.56	57.44	306	-
Model 12	743	1571	4122	1550	3399	1185	834	2898	18042	2328	5533	38148	9480	10167	61.85	38.15	227	-
Model 13	578	1283	2442	1497	2929	1340	876	20454	7278	3418	3175	15578	20097	19055	79.90	20.10	302	-
Model 14	775	1684	2444	1548	2398	1141	822	20427	7518	3427	3029	12597	14369	27821	72.18	27.82	259	-

Table S8. Confusion matrix and model selection results for the comparison between the two best-fitting models identified with the step-wise and simultaneous models selection approach using the 'modified key tooth' dataset. The most supported model is highlighted in bold.

Confusion Matrix					Model Selection	
	Model 5	Models 6	Classification Error (%)	True Positives (%)	Votes	Posterior Probability
**Model 5**	**95774**	**4226**	**4.20**	**95.80**	**602**	**76%**
Model 6	4974	95026	4.90	95.10	398	-

Table S9. Dental trait sample sizes for the ‘key tooth’ dataset. Shown are 20 ASUDAS dental non-metric traits, the observable trait sample size (n), the number of traits scored as absent (–), the number of traits scored as present (+), and the frequencies of traits scored as present (%) for six spatiotemporal groups defined for demographic modeling. For the meaning of trait abbreviations, refer to Table S1. ‘NA’ indicates the inability to calculate trait frequencies because the trait could not be observed in the spatiotemporal group. The six groups are defined as follows: West=extending from present-day Portugal to Germany; East=extending from present-day Italy to Western Russia; MPG=Middle Pleniglacial (~47–28 kya); LPG=Late Pleniglacial (~28–14.7 kya); LG&EH=Late Glacial to Early Holocene (~14.7-7 kya).

Trait	West MPG				West LPG				West LG&EH				East MPG				East LPG				East LG&EH			
	n	-	+	%	n	-	+	%	n	-	+	%	n	-	+	%	n	-	+	%	n	-	+	%
WING	0	0	0	NA	6	6	0	0.00	15	13	2	13.33	15	15	0	0.00	4	4	0	0.00	4	4	0	0.00
SHOV	8	8	0	0.00	14	11	3	21.43	13	10	3	23.08	13	11	2	15.38	2	2	0	0.00	3	1	2	66.67
DOSH	9	9	0	0.00	13	13	0	0.00	23	21	2	8.70	14	14	0	0.00	2	2	0	0.00	2	2	0	0.00
INGR	4	4	0	0.00	9	7	2	22.22	43	36	7	16.28	10	7	3	30.00	0	0	0	NA	5	4	1	20.00
MERI	2	2	0	0.00	9	7	2	22.22	22	21	1	4.55	9	7	2	22.22	0	0	0	NA	2	2	0	0.00
DARI	2	1	1	50.00	8	4	4	50.00	17	15	2	11.76	7	5	2	28.57	0	0	0	NA	1	1	0	0.00
ODON	8	8	0	0.00	18	18	0	0.00	46	46	0	0.00	14	14	0	0.00	4	4	0	0.00	7	7	0	0.00
CARA	3	2	1	33.33	21	17	4	19.05	33	25	8	24.24	19	11	8	42.11	2	1	1	50.00	8	6	2	25.00
ENEX	7	5	2	28.57	15	13	2	13.33	35	33	2	5.71	19	19	0	0.00	1	1	0	0.00	6	6	0	0.00
RUP1	5	2	3	60.00	9	2	7	77.78	5	2	3	60.00	8	1	7	87.50	0	0	0	NA	4	3	1	25.00
RUM2	5	1	4	80.00	10	5	5	50.00	11	6	5	45.45	6	1	5	83.33	0	0	0	NA	2	2	0	0.00
ANFO	10	4	6	60.00	17	7	10	58.82	7	4	3	42.86	11	5	6	54.55	3	2	1	33.33	4	2	2	50.00
DEWR	8	7	1	12.50	13	11	2	15.38	13	10	3	23.08	10	7	3	30.00	2	2	0	0.00	3	3	0	0.00
GRPA	7	5	2	28.57	19	13	6	31.58	51	42	9	17.65	24	15	9	37.50	6	6	0	0.00	17	13	4	23.53
HLM1	11	1	10	90.91	31	1	30	96.77	72	4	68	94.44	28	3	25	89.29	5	1	4	80.00	11	1	10	90.91
CUS6	10	9	1	10.00	22	18	4	18.18	64	61	3	4.69	20	18	2	10.00	5	5	0	0.00	6	5	1	16.67
CUS7	11	11	0	0.00	25	23	2	8.00	79	79	0	0.00	22	22	0	0.00	5	5	0	0.00	9	9	0	0.00
RLM1	5	5	0	0.00	26	24	2	7.69	16	16	0	0.00	13	13	0	0.00	5	5	0	0.00	4	4	0	0.00
RLM2	6	1	5	83.33	15	3	12	80.00	11	3	8	72.73	12	2	10	83.33	3	1	2	66.67	6	1	5	83.33
TOAN	0	0	0	NA	4	4	0	0.00	44	39	5	11.36	17	17	0	0.00	6	5	1	16.67	9	8	1	11.11

Table S10. Confusion matrix and model selection results for the 'Continuity' set of models using the 'key tooth' dataset. The most supported model is highlighted in bold.

Confusion Matrix						Model Selection	
	Model 1	Model 2	Model 3	Classification Error (%)	True Positives (%)	Votes	Posterior Probability
Model 1	59969	29146	10885	40.03	59.97	275	-
Model 2	46955	42605	10440	57.40	42.61	272	-
**Model 3**	**2194**	**2489**	**95317**	**4.68**	**95.32**	**453**	**50%**

Table S11. Confusion matrix and model selection results for the 'Discontinuity in the West' set of models using the 'key tooth' dataset. The most supported model is highlighted in bold.

Confusion Matrix							Model Selection	
	Model 4	Model 5	Model 6	Model 7	Classification Error (%)	True Positives (%)	Votes	Posterior Probability
Model 4	85573	2318	8823	3286	14.42	85.58	240	-
**Model 5**	**280**	**96753**	**1285**	**1682**	**3.24**	**96.76**	**262**	**59%**
Model 6	18458	7348	34820	39374	65.47	34.53	260	-
Model 7	11785	7718	28025	52472	47.52	52.48	238	-

Table S12. Confusion matrix and model selection results for the 'Discontinuity in the East' set of models using the 'key tooth' dataset. The most supported model is highlighted in bold.

Confusion Matrix										Model Selection	
	Model 8	Model 9	Model 10	Model 11	Model 12	Model 13	Model 14	Classification Error (%)	True Positives (%)	Votes	Posterior Probability
**Model 8**	**78474**	**2953**	**6773**	**2669**	**638**	**4326**	**4167**	**21.53**	**78.47**	**175**	**43%**
Model 9	7776	62495	9570	5477	9938	2335	2409	37.44	62.56	120	-
Model 10	38262	27506	16051	4109	4747	4581	4744	83.45	16.55	147	-
Model 11	590	1908	1226	91206	2262	1238	1570	8.17	91.83	173	-
Model 12	3127	19114	3169	14820	39619	10688	9463	60.59	39.41	133	-
Model 13	17258	8448	4570	10665	16667	23513	18879	76.83	23.17	134	-
Model 14	17301	9000	4575	10643	14584	17254	26643	73.91	26.09	118	-

Table S13. Confusion matrix and model selection results for the final comparison among the three best-fitting models using the 'key tooth' dataset. The most supported model is highlighted in bold.

Confusion Matrix						Model Selection	
	Model 3	Model 5	Model 8	Classification Error (%)	True Positives (%)	Votes	Posterior Probability
Model 3	73235	22407	4358	26.77	73.24	354	-
**Model 5**	**22299**	**73251**	**4450**	**26.75**	**73.25**	**385**	**69%**
Model 8	2085	1865	96050	3.95	96.05	261	-

Table S14. Confusion matrix and model selection results for the simultaneous model comparison using the 'key tooth' dataset. The most supported model is highlighted in bold.

	Confusion Matrix																Model Selection	
	Model 1	Model 2	Model 3	Model 4	Model 5	Model 6	Model 7	Model 8	Model 9	Model 10	Model 11	Model 12	Model 13	Model 14	Classification Error (%)	True Positives (%)	Votes	Posterior Probability
Model 1	61099	30086	3279	542	497	410	534	336	873	456	561	520	290	517	38.90	61.10	422	-
Model 2	48882	42099	3344	485	533	419	513	241	854	370	863	655	256	486	57.90	42.10	386	-
Model 3	3601	4352	40401	2470	17650	1994	1437	1016	3467	1417	18594	1980	758	863	59.60	40.40	423	-
Model 4	13	45	207	85013	473	9461	3805	138	166	121	195	106	137	120	14.99	85.01	408	-
Model 5	152	381	9833	496	62978	3292	4956	944	1954	951	11357	1335	775	596	37.02	62.98	405	-
**Model 6**	**11**	**64**	**157**	**23394**	**3401**	**34248**	**37708**	**164**	**147**	**147**	**162**	**137**	**142**	**118**	**65.75**	**34.25**	**442**	**42%**
Model 7	14	65	118	15920	3651	29274	50006	157	147	138	150	122	140	98	49.99	50.01	418	-
Model 8	694	1349	680	1271	1283	1467	1052	79655	1714	4009	366	598	2780	3082	20.35	79.66	391	-
Model 9	1381	2242	3815	1298	2283	1254	900	7244	56387	7046	1080	10715	2035	2320	43.61	56.39	247	-
Model 10	1056	1851	2218	1359	2329	1327	960	40181	23507	11452	696	4978	3681	4405	88.55	11.45	336	-
Model 11	1164	2117	14906	1924	10989	1701	1082	404	1373	468	57253	3291	1372	1956	42.75	57.25	304	-
Model 12	783	1604	4363	1294	3290	1241	856	3026	19341	2263	5484	38324	9430	8701	61.68	38.32	285	-
Model 13	635	1284	2561	1263	2989	1539	1003	21657	7967	3446	3214	15674	20001	16767	80.00	20.00	293	-
Model 14	840	1624	2812	1353	2368	1241	896	22042	8415	3431	3178	13159	14204	24437	75.56	24.44	240	-

Table S15. Confusion matrix and model selection results for the comparison between the two best-fitting models identified with the step-wise and simultaneous models selection approach using the 'key tooth' dataset. The most supported model is highlighted in bold.

Confusion Matrix					Model Selection	
	Model 5	Models 6	Classification Error (%)	True Positives (%)	Votes	Posterior Probability
**Model 5**	**95822**	4178	**4.10**	**95.90**	**557**	**75%**
Model 6	4469	95531	4.10	95.90	443	-

Data S1. (separate Excel file). Dental specimens and traits used for analysis.
